# Beyond standardized measures: a mixed-methods pilot study of integrated non-cognitive skills training in German upper secondary students

**DOI:** 10.3389/fpsyg.2026.1918459

**Published:** 2026-08-26

**Authors:** Sandy Schammler, Malte Persike, Franziska Weber

**Affiliations:** 1 Center für Lehr- und Lernservices (CLS), RWTH Aachen University, Aachen, Germany; 2 Städtisches Gymnasium St. Leonhard, Aachen, Germany

**Keywords:** grit, growth mindset, Kirkpatrick evaluation, mixed methods, non-cognitive skills, pilot study, School-based intervention, self-efficacy

## Abstract

**Introduction:**

Non-cognitive skills such as growth mindset, grit, self-discipline, self-efficacy, and resilience have been identified as modifiable psychological resources supporting academic functioning, yet few interventions address multiple non-cognitive skills simultaneously and evidence for integrated programs remains limited. This pilot study examined the feasibility and preliminary effects of a 1-day training addressing all five constructs among German upper secondary school students across Kirkpatrick's four evaluation levels.

**Methods:**

Using a quasi-experimental mixed-methods design, *n*_EG_ = 41 eleventh-graders participated in a 1-day, six-lesson training; *n*_CG_ = 46 served as controls. Quantitative measures included five self-report scales, a knowledge test, grade records, and 3-month transfer ratings (*n*_FU_ = 17). Immediately after the training, all *n*_EG_ = 41 participants were invited to volunteer for an interview; 10 volunteers were randomly selected and interviewed 1 month later. All had completed the pretest and posttest assessments.

**Results:**

Participants rated the training positively and showed high knowledge scores at posttest; ratings of perceived application and usefulness were substantially lower at follow-up than at posttest, although the two administrations differed in framing; and neither the targeted constructs nor academic grades changed significantly, consistent with limited statistical power. Qualitative findings extended these results: participants reported deeper conceptual learning, revised attributional beliefs, and selectively maintained self-regulatory strategies.

**Discussion:**

Overall, integrated non-cognitive skills training proved feasible within the regular school schedule, but qualitative and quantitative data yielded partially different conclusions, with interviews revealing continued strategy use and conceptual learning that standardized measures did not capture. The pronounced decline in perceived transfer highlights the need for booster sessions or curricular integration to sustain effects, and the observed effect sizes provide a foundation for planning adequately powered confirmatory trials.

## 1 Introduction

Understanding why some students thrive academically while others struggle remains a central concern in educational research. Academic success is increasingly conceptualized not merely as performance on grades or test scores but as a broader psychological capacity to pursue academic goals and cope effectively with academic challenges (Anwar et al., 2024). This perspective aligns with Dweck's conceptualization of success as a continuous process of effortful learning and personal development, in which realizing one's potential takes precedence over static performance outcomes (Dweck, 2006, 2017).

Research indicates that non-cognitive characteristics, including motivational orientations, self-regulatory capacities, and personality-related dispositions, contribute to academic performance independently of cognitive ability and become increasingly relevant over the course of schooling (Malanchini et al., 2024). Although cognitive ability remains an important predictor of educational outcomes (Kuncel and Hezlett, 2010), non-cognitive skills have been shown to predict educational attainment and labor-market success beyond intelligence quotient (IQ) and are only weakly related to cognitive ability (Heckman and Rubinstein, 2001; Smithers et al., 2018). Reflecting this growing recognition, educational policy scholars have called for integrating non-cognitive competencies more systematically into educational practice and intervention design (Heckman and Kautz, 2012; Zhao et al., 2021).

Among the non-cognitive resources garnering increased attention, (i) growth mindset (Dweck, 2006), (ii) grit (Duckworth et al., 2007), (iii) self-discipline (Duckworth and Seligman, 2005), (iv) self-efficacy (Bandura, 1977), and (v) resilience (Rutter, 2011) have been identified as modifiable psychological characteristics that shape students' academic functioning (see Sections 2.2–2.6 for definitions). These constructs capture how students interpret academic demands, regulate effort, persist through setbacks, and maintain motivation under evaluative pressure (Bandura, 1977; Duckworth et al., 2007; Dweck, 2006; Rutter, 2011). Each of these resources has been linked to academic engagement and achievement, raising the question of whether they could serve as promising targets for school-based interventions aimed at improving students' academic development (Anwar et al., 2024; Fredricks et al., 2004).

In recent years, a number of brief, low-cost intervention formats, such as 45- to 60-min growth-mindset workshops and online modules (DeBacker et al., 2018; Paunesku et al., 2015) or short-term self-regulation and cognitive modeling trainings (Mahatmaharti et al., 2019; Polirstok, 2017), have been developed and implemented in school settings to promote these resources among middle and high school students. Yet despite the growing prominence of non-cognitive skills in educational discourse, the evidence base for these interventions remains limited and heterogeneous. Reported intervention effects are often modest, vary substantially across educational contexts, and appear to depend on both individual and situational factors (Burgoyne et al., 2018; Sisk et al., 2018; Yeager and Walton, 2011). Moreover, most existing studies target isolated constructs rather than examining how multiple non-cognitive skills can be addressed within a single, integrated intervention framework. Against this background, the present study examines a school-based training program targeting growth mindset, grit, self-discipline, self-efficacy, and resilience in secondary school students. Using a quasi-experimental mixed-methods design, this pilot study evaluates the program across multiple outcome levels following Kirkpatrick's four-level evaluation framework (Kirkpatrick and Kirkpatrick, 2006). By combining standardized self-report scales, qualitative interviews, and objective academic records, the study examines not only whether the training produces effects at each evaluation level but also where different data sources converge and where they diverge.

## 2 Review of prior research

### 2.1 Non-cognitive skills as targets for school-based intervention

Academic success cannot be adequately explained by cognitive ability alone (Duckworth and Seligman, 2005; Heckman and Rubinstein, 2001; Rosen et al., 2010). Instead, empirical evidence highlights the substantial impact of specific non-cognitive resources on school performance: for instance, self-discipline has been shown to account for more than twice as much variance in adolescents' final grades as measured IQ (Duckworth and Seligman, 2005). Similarly, students' self-beliefs (particularly their confidence and domain-specific ability self-concepts) are exceptionally strong predictors of academic achievement, sometimes even outweighing corresponding intelligence scores (Stankov and Lee, 2014; Steinmayr et al., 2019). Given the profound impact of these malleable traits, Heckman and Kautz (2012) argue that interventions targeting personality-related and self-regulatory characteristics must be integral components of effective educational policy. Comprehensive meta-analytic evidence supports this position: universal school-based social and emotional learning programs significantly improve both non-cognitive competencies and academic performance, with an average 11-percentile-point gain in student achievement (Durlak et al., 2011).

Building on this rationale, the following sections address five non-cognitive resources, growth mindset, grit, self-discipline, self-efficacy, and resilience, that are examined in the present study as modifiable targets for school-based intervention.

### 2.2 Growth mindset

Growth mindset refers to the belief that intellectual abilities are malleable and can be developed through sustained effort, effective strategies, and learning from feedback (Dweck, 2006, 2017). In contrast, a fixed mindset reflects the assumption that intelligence is innate and largely unchangeable, leading students to interpret difficulty as evidence of limited ability (Dweck and Leggett, 1988). Although cognitive ability is substantially heritable and temporally stable (Plomin and Deary, 2015) it is neither fixed nor impervious to environmental influences (Nisbett et al., 2012). The growth mindset framework therefore emphasizes the extent to which students perceive their abilities as developable, because such beliefs shape academic behavior regardless of the objective stability of intelligence. Growth mindset functions as a meaning system organizing students' interpretations of academic experiences: students holding incremental views of intelligence tend to perceive challenges as developmental opportunities, interpret effort as instrumental, and construe setbacks as informative rather than threatening (Dweck, 1999).

Empirical research shows that students' implicit theories of intelligence shape their goal orientations and behavioral responses to challenge. Dweck and Leggett (1988) showed that students exposed to incremental theories adopted learning goals and persisted longer when tasks grew difficult whereas those holding entity beliefs, that is, the view that intelligence is a fixed quantity, favored performance goals and tended to disengage after failure. Growth mindset beliefs can also buffer against perceptions of task-related costs by reframing effort as valuable rather than as an indicator of low ability (Blackwell et al., 2007; Eccles, 2009).

Beyond its motivational consequences, growth mindset is a developable disposition. Longitudinal and experimental studies indicate that students' beliefs about intelligence can change over time and are shaped by learning experiences and educational contexts (Blackwell et al., 2007; Kamins and Dweck, 1999). However, the evidence on mindset interventions is mixed. More recent meta-analytic evidence corroborates this pattern, indicating that the average effect of growth mindset interventions on academic achievement tends to be very small and is moderated by the methodological rigor of the primary studies, with more rigorous studies reporting smaller effects (Macnamara and Burgoyne, 2023). Large-scale studies indicate that supportive learning environments are often necessary for belief shifts to translate into observable improvements in engagement or achievement (Yeager et al., 2016b; Yeager et al., 2022). Growth mindset is therefore best understood as a distal psychological resource: it shapes how students interpret academic demands and lays the groundwork for more proximal processes such as self-efficacy beliefs, self-disciplined behavior, and sustained effort. In the present study, growth mindset is accordingly included as a foundational component of the intervention, with the expectation that fostering incremental beliefs about intelligence may facilitate the development of the more action-oriented skills addressed in subsequent training modules (see Sections 2.3–2.6).

### 2.3 Grit

Grit is defined as the combination of passion and perseverance for long-term goals, reflecting individuals' capacity to sustain both interest and effort over extended periods despite setbacks (Duckworth and Gross, 2014; Duckworth et al., 2007). Conceptually, grit comprises two facets (perseverance of effort and consistency of interest), both regarded as central to long-term goal pursuit in educational contexts (Schmidt, 2021).

Within school settings, grit has been associated with academic engagement and persistence under demanding learning conditions. Students with higher grit are more likely to remain engaged in academic tasks and persist throughout secondary education (Eskreis-Winkler et al., 2014). The association between grit and engagement appears to be partly mediated by self-regulatory processes, as students high in grit report more frequent use of self-regulated learning strategies including planning, monitoring, and reflection (Wolters and Hussain, 2015).

At the same time, the construct has been subject to substantial conceptual debate. The central objection concerns its relation to conscientiousness, the Big Five trait domain describing a dispositional tendency toward orderliness, industriousness, self-control, and reliable goal pursuit (Roberts et al., 2014). In a meta-analytic synthesis of the grit literature, Credé et al. (2017) found overall grit to be very strongly correlated with conscientiousness and to explain essentially no variance in academic performance once conscientiousness was controlled. This conclusion did not hold uniformly across facets, however: the perseverance facet retained incremental predictive validity for grade criteria. The distinction matters here because the scale used in the present study assesses perseverance rather than the two-facet composite (see Section 3.2.1.2). Reviewing this evidence for educational contexts, Credé (2018) concluded that the case for grit as a distinct and practically useful predictor of academic success is weaker than its prominence suggests. Conscientiousness was not assessed in the present study and is referred to here only as the reference point against which the distinctiveness of grit has been contested; grit is treated throughout as the sustained pursuit of self-relevant long-term goals rather than as a general dispositional tendency. Moreover, scholars argue that sustained effort without clear goals or adaptive learning strategies may not translate into substantive academic progress (Buzzetto-Hollywood et al., 2019). Despite these criticisms, grit remains a widely studied motivational construct in educational psychology. Within the distal–proximal framework outlined above, grit occupies an intermediate position: it is more action-oriented than growth mindset, as it captures sustained behavioral engagement rather than beliefs alone, yet less situation-specific than self-discipline, which operates at the level of moment-to-moment impulse regulation (see Section 2.4). Its emphasis on long-term perseverance provides a conceptual link between growth-oriented beliefs and sustained academic effort. Consistent with this view, students who endorse a growth mindset tend to report higher levels of grit, suggesting that incremental beliefs about ability may support the motivational persistence that grit reflects (Burgoyne et al., 2018).

### 2.4 Self-discipline

Self-discipline refers to the capacity to regulate emotions, thoughts, and behaviors in accordance with self-set goals, including the ability to tolerate delayed gratification and to align everyday behavior with longer-term objectives (Duckworth, 2009; Duckworth and Seligman, 2005). In educational contexts, self-discipline supports the continued enactment of goal-directed learning behaviors, particularly when distractions or temptations threaten task focus and persistence (Duckworth, 2009).

Theoretically, self-discipline links to academic engagement through self-control processes. Self-control enables individuals to override short-term impulses in the service of higher-order goals (Baumeister and Exline, 2000), and self-discipline is the deliberate use of this capacity in academic settings. This regulatory function is particularly salient in adolescence: although future-oriented goal pursuit is often challenging at this developmental stage, even simple self-regulation strategies can substantially improve goal attainment (Duckworth et al., 2011; Romer et al., 2010). Empirically, self-discipline has been shown to predict academic performance and grade improvement even after controlling for cognitive ability (Duckworth and Seligman, 2005; Ning and Downing, 2010).

As noted above (see Section 2.3), self-discipline and grit are conceptually related yet functionally distinguishable (Hagger and Hamilton, 2019; Hwang et al., 2018). Whereas grit captures sustained perseverance toward long-term goals, self-discipline operates at the level of day-to-day behavioral regulation and resistance to short-term temptations. In the present study, self-discipline is therefore examined as a complementary, more proximal construct capturing students' situational regulatory capacity.

### 2.5 Self-efficacy

Perceived self-efficacy refers to individuals' judgments of their capabilities to organize and execute the actions required to attain specific goals (Bandura, 1977). Within social cognitive theory, self-efficacy beliefs are future-oriented, domain-specific, and assume a causal role in motivation, learning-related behavior, and performance regulation (Bandura, 1977; Zimmerman, 2000).

Self-efficacy consistently predicts academic engagement and achievement. Students with higher self-efficacy are more likely to approach challenging tasks, sustain engagement over time, and recover from setbacks, whereas students with lower self-efficacy tend to disengage from demanding learning situations (Bandura and Schunk, 1981; Zimmerman and Kitsantas, 2002). Self-efficacy explains unique variance in academic performance beyond cognitive ability and affective factors such as anxiety (Pajares and Kranzler, 1995; Vuong et al., 2010). Through self-regulatory processes such as goal setting, progress monitoring, and strategic adjustment, self-efficacy promotes sustained academic engagement and contributes to cumulative learning gains (Multon et al., 1991; Schunk, 1981; Zimmerman, 2000). In particular, self-efficacy for self-regulated learning has been identified as a key determinant of students' persistence and strategy use when confronting academic demands (Usher and Pajares, 2008).

Conceptually, self-efficacy is related to, yet distinct from, mindset beliefs: whereas growth mindset reflects generalized assumptions about the malleability of intelligence, self-efficacy refers to domain-specific beliefs about one's capability to act effectively and influence outcomes, for example, a student's confidence in being able to solve algebraic equations or to write a coherent essay (Bandura and Locke, 2003; Degol et al., 2018). Self-efficacy operates alongside self-discipline and grit in predicting academic success, with each construct contributing uniquely to students' persistence, effort regulation, and engagement (Alhadabi and Karpinski, 2020; Soruç et al., 2022). Within the distal–proximal framework adopted here, self-efficacy occupies a proximal position: it consists of future-oriented, domain-specific judgments that are formed and revised through concrete mastery experiences, and therefore responds more directly to targeted intervention than generalized beliefs about ability.

### 2.6 Resilience

Resilience is conceptualized as a context-specific process of positive adaptation to academic stressors, setbacks, and learning-related challenges (Kalisch et al., 2019; Rutter, 2011). Rather than a fixed trait, resilience is a dynamic process that enables individuals to maintain or regain effective functioning when facing adversity (Brewer et al., 2019). Within educational contexts, resilience captures students' capacity to cope adaptively with ongoing academic demands, evaluative pressure, and experiences of failure (Martin, 2002; Martin and Marsh, 2006).

Empirical research links resilience to sustained motivation, persistence, and effective learning behavior (Alva, 1991; Wang and Gordon, 1994). Resilience is positively associated with academic achievement (Ayala and Manzano, 2018) and psychological wellbeing in educational contexts (Denovan and Macaskill, 2017). Resilience is not considered an innate characteristic but a developable capacity that can be strengthened over time through targeted intervention (Luthar et al., 2000; Thomas and Asselin, 2018). Conceptually, resilience builds on the other non-cognitive resources examined in this study: the incremental beliefs fostered by a growth mindset, the long-term perseverance captured by grit, the situational regulatory capacity reflected in self-discipline, and the agentic self-beliefs central to self-efficacy. Within the distal–proximal framework adopted here, resilience can be understood as an integrative, context-dependent capacity that draws on these resources to sustain effective functioning under ongoing academic demands and setbacks (Bandura, 1995).

### 2.7 Construct overlap and integrative rationale

As outlined in the preceding sections, the five non-cognitive constructs examined here (growth mindset, grit, self-discipline, self-efficacy, and resilience) are theoretically related yet capture functionally distinguishable aspects of students' motivation and self-regulation along the distal–proximal continuum introduced above. Two complementary observations motivate addressing them within a single intervention framework.

First, each construct has been independently linked to academic engagement and achievement, suggesting that targeting multiple resources simultaneously may address a broader range of the psychological processes underlying academic functioning than single-construct approaches. Second, prior research documents moderate-to-strong intercorrelations among grit, self-discipline, self-efficacy, and resilience (Alhadabi and Karpinski, 2020; Credé, 2018), indicating that these resources do not operate in isolation but may reinforce one another, a pattern that an integrated intervention is uniquely positioned to leverage.

At the same time, these overlaps raise questions of discriminant validity that concern the five constructs themselves and not only their relation to broader personality dimensions. Three boundaries are at issue. First, growth mindset and self-efficacy are both belief constructs but differ in their object: growth mindset concerns the malleability of ability in general, whereas self-efficacy concerns students' confidence in their own capacity to organize and execute the actions required to meet academic demands (Bandura, 1977; Dweck, 1999). Second, grit and self-discipline both describe the alignment of action with intention but are situated at different levels of the goal hierarchy: self-discipline concerns the resolution of momentary conflicts between temptation and any valued goal, whereas grit concerns the sustained pursuit of a single superordinate goal over months and years (Duckworth and Gross, 2014). Third, resilience differs from the remaining four in kind rather than in degree: it is understood here as a process of positive adaptation that can only be inferred where adversity has occurred, and it draws on the other resources rather than standing alongside them (Kalisch et al., 2019; Rutter, 2011).

These boundaries are not equally secure. The distinction between grit and self-discipline is the weakest, both theoretically and empirically: meta-analytic evidence places the correlation between grit and self-control at ρ = 0.72 (Credé et al., 2017), and the two scales employed here, one of which is a self-control measure, correlated at *r* = 0.76 at baseline (see Section 4.1). The present study therefore treats this overlap as a substantive constraint on interpretation rather than as a caveat. Outcomes are reported and interpreted construct by construct rather than as a composite score; baseline intercorrelations are presented as an explicit check on discriminant validity; and findings for grit and self-discipline are interpreted jointly rather than as independent evidence. Because all five constructs were addressed within a single training day, the design supports conclusions about the integrated program as a whole and not about the contribution of individual modules.

### 2.8 Rationale for intervention-based approaches in school contexts

During adolescence, intensifying academic demands and heightened social comparison can undermine students' motivational and self-regulatory resources, contributing to disengagement, elevated stress, and reduced academic functioning (Claro et al., 2016; Yeager et al., 2019). Schools represent a critical context for interventions because they provide systematic access to students during this developmentally sensitive phase and embed learning-related challenges that naturally activate motivational and self-regulatory processes (Yeager and Walton, 2011). School-based programs also offer an efficient delivery mechanism that can reach large numbers of students at a central developmental setting (Furlong et al., 2014).

Although the evidence base for brief school-based interventions remains heterogeneous (see Section 1), a number of studies indicate that the non-cognitive skills targeted in the present study are, in principle, modifiable through structured programs. Growth mindset interventions have been shown to promote incremental beliefs about intelligence, increase effort and motivation, and improve academic outcomes, with effects that are particularly pronounced among initially lower-achieving students and those from disadvantaged backgrounds (Blackwell et al., 2007; Claro et al., 2016; Paunesku et al., 2015; Yeager et al., 2016a). Yeager et al. (2019) further demonstrated that such interventions can produce significant effects even without accompanying teacher training. Mindset-focused programs may also foster related dispositions, as research suggests that promoting growth beliefs can simultaneously enhance grit, self-efficacy, and academic performance (Claro et al., 2016; DeBacker et al., 2018; Dweck, 2018; Perkins-Gough, 2013). Recent research demonstrates that grit can indeed be cultivated through targeted educational interventions, with evidence ranging from smaller, context-specific programs (Mirza et al., 2021) to large-scale randomized controlled trials (Santos et al., 2022). For example, a nationwide intervention involving a large sample of middle-school students successfully improved self-regulation and the perseverance-of-effort facet of grit (Santos et al., 2022). Developing such non-cognitive resources is highly relevant for educational outcomes, as grit-enhancing programs hold the potential to reduce dropout rates, prevent academic maladjustment, and support long-term academic persistence (Hwang et al., 2018).

Self-efficacy beliefs are similarly responsive to intervention; modeling, targeted feedback, and mastery experiences have been identified as effective strategies for strengthening efficacy beliefs, which in turn mediate changes in self-regulation and performance outcomes (Gist and Mitchell, 1992; Li et al., 2025; Schunk, 1981; Zimmerman, 2000). For resilience, current evidence supports the potential benefits of school-based training for mental health and wellbeing (Joyce et al., 2018). Regarding self-discipline, the capacity to delay gratification has been identified as a powerful predictor of long-term academic and social outcomes (Funder et al., 1983; Mischel et al., 1989), although more recent conceptual replications suggest that the long-term predictive power of delay-of-gratification tasks diminishes substantially when broader cognitive and family-background factors are controlled (Watts et al., 2018). Nevertheless, learning self-control strategies may help reduce developmental risks such as academic failure, particularly among at-risk youth (Duckworth et al., 2011; Mischel et al., 1989).

From a psychological mechanisms perspective, brief interventions targeting non-cognitive skills operate through several complementary processes. Active reflection exercises help students reframe their experiences by emphasizing adaptive attributions and disrupting unproductive thought patterns (Walton and Wilson, 2018). Techniques that reshape students' self-identity, such as structured prompts encouraging reflection on personal goals and strengths, can motivate behavior consistent with new self-understandings (Walton and Wilson, 2018). Implementation intention strategies and autonomy-supportive approaches have additionally been shown to enhance goal attainment and motivation for behavioral change (Walton and Wilson, 2018).

Effect sizes in this literature are nevertheless modest. Kraft (2020) has argued that effects of 0.10–0.15 standard deviations should be considered meaningful when interventions are scalable, rigorously evaluated, and assessed against official outcome measures such as grades.

Against this background, the present study addresses this gap by examining a comprehensive school-based intervention that integrates five non-cognitive skills within a single training program. This pilot study aims to clarify the program's potential effectiveness and limitations, thereby contributing to a more nuanced understanding of how non-cognitive skills can be fostered in secondary school contexts. To this end, the study adopts the four-level training evaluation framework proposed by Kirkpatrick and Kirkpatrick (2006), which distinguishes four hierarchically ordered outcome dimensions: participants' immediate satisfaction with and perceived relevance of the program (Level 1: Reaction), the acquisition of knowledge and changes in attitudes (Level 2: Learning), the transfer of learned content into everyday behavior (Level 3: Behavior), and broader performance-related outcomes such as academic achievement (Level 4: Results). Originally developed for organizational training contexts, the Kirkpatrick framework has since been adopted in educational research and is particularly suited for evaluating complex interventions whose effects may manifest at some outcome levels but not at others.

The dosage of the present intervention warrants explicit consideration. Brief school-based programs targeting non-cognitive skills are typically short, ranging from single exercises of 15–20 min to sessions of about 1 h (DeBacker et al., 2018; Paunesku et al., 2015; Yeager and Walton, 2011), and their effects are theorized to operate recursively: a short intervention shifts how students interpret academic experiences, and the surrounding environment then either sustains or dampens that shift (Walton and Wilson, 2018; Yeager and Walton, 2011). At 270 min of contact time, distributed across all five constructs, the present training exceeds this range by a wide margin. Prior work on combined interventions has raised, but not resolved, the question of how dosage should be allocated across components: Paunesku et al. (2015) attributed the absence of an added benefit in their combined condition to students receiving only a partial dose of each component, and Walton and Wilson (2018) argue that timing may matter more than dosage for interventions of this kind. Dose-response relationships for programs addressing several non-cognitive constructs in a single session have not been established. Whether the present dosage is adequate, and for which constructs, is therefore among the questions this pilot evaluation is intended to inform.

### 2.9 Study objectives, research questions, and hypotheses

Given the still limited and mixed evidence regarding brief school-based interventions targeting non-cognitive skills (Burgoyne et al., 2018; Yeager and Walton, 2011), specific research questions (RQ) **or** hypotheses (H) were formulated for each of the four evaluation levels, depending on whether the existing evidence supported directional predictions. Research questions guided the exploratory analyses at Levels 1 (Reaction) and 3 (Behavior), where no directional predictions were specified in advance, whereas directional hypotheses were formulated for Levels 2 (Learning) and 4 (Results), where prior theory and evidence supported specific expectations.

#### 2.9.1 Level 1—Reaction (L1)

The first level focuses on students' immediate perceptions of the intervention, as participants' satisfaction and perceived relevance are considered important prerequisites for subsequent learning and transfer processes.

**RQ1:** How do students evaluate the intervention in terms of satisfaction, relevance, and perceived usefulness?

#### 2.9.2 Level 2—Learning (L2)

At the learning level, the intervention aims to foster students' knowledge and conceptual understanding of key non-cognitive skills (growth mindset, grit, self-discipline, self-efficacy, and resilience), which are theoretically linked to academic engagement and functioning (Anwar et al., 2024; Bandura, 1977; Duckworth et al., 2007; Dweck, 2006; Rutter, 2011).

**H1:** In the posttest, the experimental group will demonstrate topic-specific knowledge about growth mindset, grit, self-discipline, self-efficacy, and resilience that (a) exceeds chance-level responding on the domain-specific knowledge test, and (b) is reflected in qualitative feedback through deeper conceptual understanding and changes in attributional patterns.

#### 2.9.3 Level 3—Behavior (L3)

The third level examines the transfer of learned skills into everyday school life. Since non-cognitive skills are expected to support students' engagement, persistence, and coping with academic challenges (Fredricks et al., 2004; Martin and Marsh, 2006; Zimmerman, 2000), it is crucial to explore whether and how students apply the acquired skills beyond the intervention context.

**RQ2:** To what extent do participants apply the taught non-cognitive skills in their everyday school life, and which factors facilitate or hinder this transfer?

#### 2.9.4 Level 4—Results (L4)

At the outcome level, the intervention is expected to influence both academic performance and the development of the targeted non-cognitive constructs, which are widely discussed as modifiable predictors of academic functioning and success-related processes (Anwar et al., 2024; York et al., 2015).

**H2:** Grades will improve more strongly from the first to the second half of the school year in the experimental group compared to the control group.

**H3:** The targeted non-cognitive constructs (growth mindset, grit, self-discipline, self-efficacy, and resilience) will improve significantly from pretest to follow-up in the experimental group.

## 3 Methods

### 3.1 Participants

A total of *n*_*Tot*_= 87 students from the 11th grade of a German upper secondary school (Gymnasium) were eligible for participation. The experimental group (EG) consisted of *n*_*EG*_= 41 students who voluntarily enrolled in the training and for whom written parental consent was obtained. These participants completed all questionnaire-based assessments and took part in the intervention. All students in the 11th-grade cohort were eligible, and no exclusion criteria beyond cohort membership were applied. Enrolment was voluntary, so the resulting sample is a convenience sample from a single institution in which group membership was determined by students' own decision rather than by allocation.

The remaining *n*_*CG*_= 46 students from the same grade level constituted the control group (CG). These students did not participate in the intervention and did not complete questionnaire measures; only anonymized academic grades were obtained for comparative purposes. Consequently, questionnaire-based demographic information was available exclusively for the experimental group. Because students enrolled voluntarily and the control group completed no questionnaires, all analyses of psychological outcomes (Levels 1–3 and Level 4b) rest on a single-group pretest-posttest-follow-up design without a comparison condition. Academic grades were available for both groups at both time points, so the between-group comparison at Level 4a controls for stable baseline differences between the groups; it does not, however, rule out that students who chose to participate differed in their trajectory of academic change.

Participants in the experimental group had a mean age of 16.30 years (*SD* = 0.53; range: 16–18). Of these, 26 identified as female (63.4%) and 15 as male (36.6%). No participant reported a non-binary gender identity.

At the 3-month follow-up assessment, all students in the experimental group were invited to complete the questionnaire battery. 17 of the *n*_*EG*_= 41 participants returned complete follow-up data, corresponding to a response rate of 41.5%. Participation remained voluntary and was not incentivized.

A separate subsample took part in the qualitative component. Immediately after the training, all *n*_EG_ = 41 participants were invited to volunteer for an interview. 10 students were then randomly selected from those who volunteered and were interviewed approximately 1 month later. All 10 had completed the pretest and posttest assessments. Because interview data were anonymized separately from the questionnaire data, interviewees cannot be matched to the *n*_FU_ = 17 participants who provided complete follow-up data; the qualitative and follow-up samples therefore overlap to an unknown degree.

#### 3.1.1 Sensitivity analyses

Because the sample size was constrained by the number of students available in the participating classes, sensitivity analyses were conducted instead of conventional *a priori* power analyses. All sensitivity analyses were performed using G^*^Power version 3.1.9.7 (Faul et al., 2007), assuming two-tailed tests and an alpha level of 0.05.

For the comparison of academic grades between the experimental group (*n*_*EG*_= 41) and the control group (*n*_*CG*_= 46), an independent-samples *t* test with a desired power of 1 – β = 0.95 was specified. The sensitivity analysis indicated that, given the realized sample sizes, the study was adequately powered to detect large between-group effects (*d* ≥ 0.78).

A second sensitivity analysis was conducted to assess within-group change in the experimental group from pretest to follow-up. A paired-samples *t* test with a desired power of 1 – β = 0.95 was specified based on the *n*_*FU*_= 17 participants who provided complete pre–follow-up data. This analysis indicated that the study was sensitive only to large within-group effects (*d*_z_ ≥ 0.93).

Taken together, these analyses indicate that the present design was adequately powered to detect large effects, whereas small-to-moderate effects may not have been detectable with the available sample sizes. The same constraint applies to the precision of the parameter estimates themselves: means, correlations, and reliability coefficients derived from these samples carry wide confidence intervals and should be read as provisional. Effect sizes are therefore reported throughout and interpreted as the primary indicators of change magnitude, with significance tests treated as secondary. This limitation is addressed in the Discussion.

### 3.2 Instruments

The study employed a mixed-methods assessment strategy integrating standardized self-report questionnaires, a researcher-developed knowledge test, self-constructed evaluation measures, and semi-structured interviews. The instruments are described in more detail below.

#### 3.2.1 Standardized self-report scales

The quantitative component comprised five validated German-language self-report scales assessing growth mindset, grit, self-discipline, general self-efficacy, and resilience. Some instruments were originally developed in English and subsequently translated and psychometrically validated for German-speaking populations by independent research teams, whereas others were originally developed and validated in German. Each scale employed Likert-type response formats and has demonstrated acceptable reliability and construct validity in prior research. Because reliability coefficients estimated in samples of this size carry considerable uncertainty, 95% confidence intervals are reported alongside each α (Feldt, 1965); these values describe the present sample and are not intended as independent validation of the instruments. No study-specific factor analyses were conducted, because a sample of *n*_EG_ = 41 does not permit stable estimation of measurement models; the psychometric evidence for these instruments therefore rests on the published validation studies cited above.

A domain-specific knowledge test was developed to assess learning outcomes related to the instructional content of the training (Level 2: Learning). Item construction followed predefined learning objectives, and all items were reviewed for alignment with the targeted competencies. Prior to the main study, items were pilot-tested with non-participating students to evaluate item difficulty and comprehensibility. Minor linguistic adjustments were implemented based on pilot feedback. The complete item set is provided in Supplementary Table 3.

To assess training-related perceptions and self-reported behavioral intentions, a brief reaction and transfer questionnaire was developed in accordance with the evaluation framework proposed by Kirkpatrick and Kirkpatrick (2006). The instrument included items capturing perceived relevance, satisfaction, and intention to apply the strategies addressed during the training, corresponding to Level 1 (Reaction) and Level 3 (Behavior).

The qualitative component consisted of semi-structured interviews guided by a protocol aligned with the four-level evaluation model of Kirkpatrick and Kirkpatrick (2006). The interview guide was designed to explore participants' reactions to the training, perceived learning gains, behavioral transfer, and perceived outcomes, while allowing flexibility to capture individual experiences in depth.

##### 3.2.1.1 Growth mindset

Growth mindset was assessed using the German adaptation of the three-item Implicit Theories of Intelligence Scale (Dweck et al., 1995). The instrument captures beliefs about the malleability of intelligence and distinguishes between entity (fixed) and incremental (malleable) conceptions of intellectual ability. The German version applied in this study is the validated adaptation by (Rammstedt et al., 2024), which was translated using the TRAPD approach (Translation, Review, Adjudication, Pretesting, Documentation) and psychometrically validated for adolescent and adult populations.

The scale consists of three statements reflecting a fixed mindset, such as “You have a certain amount of intelligence, and you really cannot do much to change it.” Participants rated their agreement on a 6-point Likert scale ranging from 1 (strongly agree) to 6 (strongly disagree). All items were reverse-coded so that higher scores reflect stronger endorsement of a growth mindset. A composite score was computed by averaging the three items.

The German adaptation has demonstrated acceptable internal consistency in previous validation studies (α = 0.83–0.90; Rammstedt et al., 2024). In the present sample, internal consistency was α =0.90, 95% CI (0.83, 0.94).

##### 3.2.1.2 Grit

Grit was assessed using the German adaptation of the Grit Scale (Duckworth et al., 2007), validated within the GESIS ZIS framework by Breyer and Danner (2015). The nine-item version represents a psychometrically evaluated unidimensional measure of perseverance toward long-term goals. Participants rated each item on a 5-point Likert scale ranging from 1 (“not at all”) to 5 (“to a very high extent”). Negatively keyed items were reverse-scored, and a mean composite score was computed, with higher values reflecting greater grit. A representative item is: “I finish whatever I begin.”

The German validation study reported acceptable internal consistency (Cronbach's α = 0.62–0.74; Breyer and Danner, 2015). In the present study, internal consistency was α = 0.73, 95% CI (0.59, 0.84).

##### 3.2.1.3 Self-discipline

Self-discipline was assessed using the German short version of the Self-Control Scale (SCS-K-D; Bertrams and Dickhäuser, 2009). The scale consists of 13 items assessing perceived self-discipline and self-regulatory capacity. Responses were recorded on a 5-point Likert scale ranging from 1 (strongly disagree) to 5 (strongly agree). Negatively keyed items were reverse-coded, and an overall self-discipline score was computed by aggregating item responses, with higher scores indicating greater dispositional self-discipline.

The German short version has demonstrated good internal consistency in student samples, including secondary school students (Cronbach's α ≈ 0.85; Bertrams and Dickhäuser, 2009). In the current study, internal consistency was acceptable [Cronbach's α = 0.65, 95% CI (0.48, 0.79)].

##### 3.2.1.4 Self-efficacy

General self-efficacy was assessed using the General Self-Efficacy Scale (GSES; Schwarzer and Jerusalem, 2003). The GSES consists of 10 items designed to capture optimistic self-beliefs regarding perceived competence in dealing with difficulties and barriers in everyday life. Items are rated on a 4-point Likert scale ranging from 1 (not at all true) to 4 (exactly true). A mean score across all 10 items was computed (possible range 1–4), with higher scores indicating stronger general self-efficacy beliefs.

The original German version was administered in this study. This version has been extensively validated and normed in a large, population-representative German sample and demonstrated excellent internal consistency (Cronbach's α = 0.92; Hinz et al., 2006). In the current sample, internal consistency was good [Cronbach's α = 0.83, 95% CI (0.75, 0.90)].

##### 3.2.1.5 Resilience

Resilience was assessed using the Brief Resilience Scale (BRS; Smith et al., 2008). The BRS consists of six items designed to capture the capacity to recover from stressful experiences. Items are rated on a 5-point Likert scale ranging from 1 (strongly disagree) to 5 (strongly agree). Following established scoring procedures, negatively worded items were reverse-coded, and a mean score across all six items was computed, with higher values indicating greater resilience.

The German version of the Brief Resilience Scale was administered. Previous research has reported good internal consistency for this instrument in a population-based German sample (Cronbach's α = 0.85; Chmitorz et al., 2018). In the current sample, internal consistency was acceptable [Cronbach's α = 0.79, 95% CI (0.67, 0.88)].

#### 3.2.2 Training evaluation

Students' immediate reactions to the workshop were assessed using three brief self-report measures developed specifically for the present study to capture key aspects of Level 1 (Reaction) training evaluation. All items were administered in German and rated on a 5-point Likert-type scale ranging from 1 (not at all) to 5 (very much). Mean scores were computed for each scale, with higher values indicating more positive evaluations. As these instruments were designed as brief post-training feedback measures rather than as psychometrically validated scales, internal consistency estimates are reported for descriptive purposes only.

Workshop satisfaction was assessed with five items capturing overall satisfaction with the workshop content, trainer, instructional design, and organizational aspects. Items addressed the quality of the content, the trainer's preparedness, the balance between theoretical input and practical exercises, the methods and activities used (e.g., group work and discussions), and the organization and scheduling of the session. Internal consistency in the present sample was acceptable (Cronbach's α = 0.73).

Perceived application was measured with three items assessing the perceived applicability and motivational impact of the workshop for students' academic everyday life. These items addressed the relevance of the training for current academic challenges, its motivational value for skill development, and its perceived contribution to academic performance. Internal consistency was acceptable (Cronbach's α = 0.71).

Perceived usefulness of the trained non-cognitive skills was assessed with five items evaluating how helpful each core topic (growth mindset, grit, self-discipline, self-efficacy, and resilience) was perceived to be for students' personal development and academic performance. This scale demonstrated good internal consistency (Cronbach's α = 0.84).

At the 3-month follow-up (*n*_FU_ = 17), internal consistency for the modified scales was good (perceived application: Cronbach's α = 0.86; perceived usefulness: Cronbach's α = 0.91). The posttest and follow-up versions of these scales were not identical in content. Posttest items asked participants to rate their expectation that the training would be applicable and useful, whereas follow-up items asked them to report retrospectively whether they had applied the contents and how useful the topics had been over the preceding 3 months. Posttest-to-follow-up comparisons on these scales therefore reflect a shift from prospective expectation to retrospective report in addition to any change in perceived transfer.

The original German item wording and corresponding English translations are provided in Supplementary Tables 1, 2.

### 3.3 Procedures

Data collection was conducted in spring 2025 at a German upper secondary school (Gymnasium) and followed the four-level evaluation framework proposed by Kirkpatrick and Kirkpatrick (2006).

After approval by the school administration, all 11th-grade students were informed about the training during regular school hours. Participation was voluntary and required written informed consent from both students and their legal guardians. Group allocation followed a self-selection process, reflecting the quasi-experimental nature of the study.

The intervention was implemented as a 1-day training during the regular school schedule at the beginning of the second academic term, comprising six consecutive school lessons of 45 min each. The training addressed five psychosocial competencies (growth mindset, grit, self-discipline, self-efficacy, and resilience) and followed a standardized session structure combining theoretical input, applied exercises, and guided group discussions. The five constructs were introduced in a deliberate sequence, beginning with growth mindset as the belief-level foundation and progressing toward increasingly action-oriented content, with cross-references between modules made explicit throughout. Each construct was addressed through instructional input, at least one applied individual exercise, and guided discussion, but the instructional mechanisms differed across constructs. Growth mindset was addressed primarily through conceptual reframing supported by neurobiological explanation and autobiographical reflection. Self-discipline was operationalized through mental contrasting with implementation intentions, and self-efficacy through a structured goal-setting protocol combining proximal sub-goals with a distal target; participants applied both protocols to self-selected personal goals during the session. Grit was addressed through a four-component framework covering interest, deliberate practice, purpose, and hope, together with a reflection task on personal goals and their perceived meaning, and resilience through reflective exercises on personal energy resources and internalized performance standards. A shared module on evidence-based learning strategies, including distributed practice and retrieval practice, provided cross-cutting techniques applicable across all five domains.

Quantitative data collection within the experimental group occurred at three measurement points. At pretest (T1), conducted immediately prior to the intervention, participants completed the five self-report questionnaires assessing growth mindset, grit, self-discipline, self-efficacy, and resilience. At posttest (T2), administered directly after completion of the final training session, participants completed a domain-specific knowledge test assessing learning outcomes (Level 2: Learning) as well as reaction items evaluating immediate training perceptions (Level 1: Reaction). The five non-cognitive skill scales were not readministered at T2; they were assessed at T1 and T3 only. At follow-up (T3), approximately 3 months after the intervention, participants again completed the five self-report questionnaires assessing the target non-cognitive skills, accompanied by modified reaction items capturing perceived behavioral transfer and application in everyday school life (Level 3: Behavior). Pretest and posttest were completed on site during school hours, with participants accessing the online survey via a QR code, whereas the follow-up battery was distributed through the school's learning management system and completed without supervision; two reminders were sent at two-week intervals.

To complement the quantitative data, a qualitative interview phase was conducted approximately 1 month after the intervention. All participants in the experimental group had been invited to volunteer for an interview immediately after the training, and 10 students were randomly selected from those who volunteered. Semi-structured interviews were conducted on school premises, lasted 30–45 min, and were audio-recorded with consent. All interviews were transcribed verbatim and anonymized prior to analysis.

Academic outcomes (Level 4: Results) were assessed using official school grades obtained for two grading periods within the same academic year. Grades from the first school term served as a pre-intervention baseline indicator of academic performance, whereas grades from the second school term represented post-intervention academic outcomes. The intervention was implemented a few weeks after the beginning of the second term, ensuring that the first-term grades were unaffected by the training. All grade data were provided by the school administration in pseudonymized form and were matched to participants' questionnaire data using a coded identifier, so that individual-level linkage was possible without revealing students' identities to the research team.

### 3.4 Data analysis

Quantitative and qualitative data were analyzed in accordance with the study's mixed-methods design and the four-level evaluation framework proposed by Kirkpatrick and Kirkpatrick (2006). All quantitative analyses were conducted using R (version 4.5.3) (R Core Team, 2026); analysis script development was supported by Claude (claude-opus-4-6; Anthropic, PBC, San Francisco, CA, United States). Statistical significance was evaluated at a two-sided alpha level of 0.05.

#### 3.4.1 Quantitative analysis

After descriptive analyses, to evaluate Level 1 (Reaction; i.e., participants' immediate evaluations of the training), one-sample *t* tests compared post-intervention evaluation scores against the neutral midpoint of the response scale. Effect sizes were reported as Cohen's *d*.

For Level 2 (Learning; i.e., acquisition of topic-specific knowledge), the domain-specific knowledge test comprised 10 item sets, each presenting five statements to be judged as true or false, yielding 50 binary items scored as the proportion correct. Because each statement required a dichotomous judgment, the theoretical chance level was 0.50. Performance was analyzed using a one-sample *t* test against this value. Internal consistency of the knowledge test was assessed using Cronbach's α.

Analyses of Level 3 (Behavior; i.e., transfer of trained skills to everyday school life) focused on participants with complete data at both posttest and follow-up. One-sample *t* tests evaluated perceived transfer relative to the scale midpoint, and paired-samples *t* tests examined changes from posttest to follow-up. Effect sizes for within-person comparisons were reported as Cohen's *d* for within-person change (*d*_*z*_). As follow-up questionnaires contained no partially missing responses, analyses were based on complete cases.

To assess potential selective attrition, participants with and without follow-up data were compared on key post-intervention variables using independent-samples *t* tests, with Hedges' *g* reported as the effect size.

For Level 4 (Results; i.e., academic outcomes), changes in academic grades from the first to the second school term were analyzed using a mixed-design analysis of variance (ANOVA), with time [first school term (Term 1) vs. second school term (Term 2)] as a within-subjects factor and group (experimental vs. control) as a between-subjects factor. Partial eta squared (η^2^_p_) was reported as the measure of effect size. To corroborate these findings, linear mixed-effects models with random intercepts for students were estimated. In addition, change-score analyses compared mean grade improvements between groups.

Also for Level 4 (Results; i.e., non-cognitive outcomes), pretest-to-follow-up changes in the five target constructs within the experimental group were examined using paired-samples *t* tests. Given the small follow-up sample (*n*_*FU*_ = 17), these analyses were treated as exploratory, and effect sizes were interpreted as the primary indicators of change magnitude. To control for multiple comparisons, *p* values were adjusted using Holm's sequential correction procedure (Holm, 1979). As a robustness check, Wilcoxon signed-rank tests were conducted to verify that results were not dependent on the normality assumption.

#### 3.4.2 Qualitative analysis

Qualitative interview data were analyzed using thematic analysis following the six-phase approach outlined by Braun and Clarke (2006). Interviews were transcribed verbatim and analyzed using MAXQDA (VERBI Software, 2026). A hybrid coding strategy combining deductive and inductive approaches was employed. Deductive coding was guided by the Kirkpatrick framework and the five target constructs (growth mindset, grit, self-discipline, self-efficacy, and resilience), while inductive coding was used to identify emergent themes related to training experiences, transfer processes, and perceived barriers or facilitators.

Two researchers independently coded all interview transcripts using MAXQDA. Initial intercoder agreement was assessed using two complementary approaches. At the document level, percentage agreement across all codes was 76.04%, indicating substantial concordance in the identification of themes within each interview. At the segment level, intercoder agreement computed with MAXQDA's intercoder function using Brennan and Prediger (1981) κ was κ =0.30, corresponding to fair agreement (Landis and Koch, 1977). The discrepancy between document-level and segment-level agreement is consistent with the use of a fine-grained coding system comprising 46 codes, where minor differences in segment boundaries reduce overlap even when coders agree on the presence and location of a theme. All coding discrepancies were subsequently resolved through iterative discussion until full consensus was achieved. Related codes were then clustered into overarching themes, which were iteratively reviewed and refined to ensure conceptual coherence and internal consistency.

#### 3.4.3 Mixed-methods integration

Consistent with a mixed-methods design with quantitative priority (Creswell and Plano Clark, 2018), quantitative analyses were used to test the predefined hypotheses, whereas qualitative findings served to contextualize, elaborate, and substantiate the quantitative results. Integration occurred at the interpretation stage, enabling triangulation and a more nuanced understanding of the training effects across the four evaluation levels.

## 4 Results

### 4.1 Descriptive statistics and correlations

Descriptive statistics and intercorrelations for the baseline non-cognitive target constructs and baseline grades in the experimental group are presented in Table 1. Shapiro–Wilk tests indicated no significant departures from normality for the five non-cognitive baseline scales (all *W*s ≥ 0.95, all *p*s ≥ 0.052). Baseline grades deviated significantly from normality (*W* = 0.92, *p* = 0.005), consistent with the discrete nature of the German grading scale. Skewness and kurtosis values across all six baseline variables remained within conventional thresholds (|skewness| ≤ 0.74, |kurtosis| ≤ 1.00; Curran et al., 1996). Because Pearson correlations are robust to moderate non-normality at this sample size (Bishara and Hittner, 2012), parametric procedures were retained; Spearman rank correlations yielded a qualitatively identical pattern (maximum absolute difference in correlation coefficients = 0.09). At baseline, students in the experimental group reported generally moderate levels of growth mindset, grit, self-discipline, self-efficacy, and resilience, and their mean grade point average across all subjects (*M* = 10.64, *SD* = 1.87, on the German 0–15 grading scale) was in the mid-range of the scale.

**Table 1:** Descriptive statistics and Pearson correlations among baseline variables (experimental group).

Variable	*M*	*SD*	1	2	3	4	5
1. Growth mindset (pre)	3.54	0.88	–				
2. Grit (pre)	3.25	0.49	0.02	–			
3. Self-discipline (pre)	3.00	0.44	−0.03	0.76^***^	–		
4. Self-efficacy (pre)	2.78	0.43	0.26	0.45^**^	0.40^**^	–	
5. Resilience (pre)	3.42	0.74	−0.15	0.49^**^	0.30	0.53^***^	–
6. Average grade Term1	10.64	1.87	0.24	0.10	0.10	0.14	−0.15

All variables were assessed at baseline in the experimental group (*n*_*EG*_ = 41). Correlations are shown below the diagonal. ^**^p < 0.01, ^***^p < 0.001.

Exploratory Pearson correlations among these variables are also shown in Table 1. The non-cognitive constructs were moderately to strongly intercorrelated, particularly the combinations involving grit, self-discipline, self-efficacy, and resilience. Following the discriminant validity considerations set out in Section 2.7, these correlations were inspected as a check on whether the five scales capture separable constructs in the present sample. Grit and self-discipline correlated at *r* = 0.76, close to the meta-analytic estimate for the relation between grit and self-control; results for this pair are therefore not treated as independent evidence. In contrast, growth mindset was only weakly associated with the other non-cognitive scales. Correlations between the non-cognitive constructs and baseline grades were small and non-significant, suggesting that motivational and self-regulatory resources were only weakly related to academic achievement at baseline. As these correlational analyses were exploratory and not part of the planned hypothesis tests, they are reported without correction for multiple testing and are interpreted descriptively.

### 4.2 Testing the hypothesized model

#### 4.2.1 Level 1—Reaction (RQ1)

Descriptive statistics showed that students evaluated the workshop positively across all three reaction scales. Mean scores were above the neutral midpoint of the scale (3) for workshop satisfaction [*M* = 4.03, *SD* = 0.57, 95% CI (3.85, 4.21)], perceived application [*M* = 3.86, *SD* = 0.61, 95% CI (3.67, 4.06)], and perceived usefulness of the trained non-cognitive skills [*M* = 3.87, *SD* = 0.65, 95% CI (3.67, 4.07)]. One-sample *t* tests against the midpoint indicated that all three means were significantly higher than 3, *t* (40) = 11.60, 8.98, and 8.62, respectively, all *p* < 0.001. The magnitude of these differences was large, with Cohen's *d* values of 1.82 for satisfaction, 1.40 for perceived application, and 1.35 for perceived usefulness. Full statistics are reported in Supplementary Table 4.

Qualitative feedback was consistent with the quantitative results. Students reported the diversity of instructional methods, the supportive learning atmosphere, and the use of metaphors as beneficial elements of the workshop. At the same time, some participants mentioned moments of perceived cognitive overload and expressed a desire for increased opportunities for group-based activities. In addition, several students described perceived increases in their academic motivation beyond the workshop context. For instance, one student reported approaching mathematics lessons with renewed effort, noting that having a new teacher provided “a new motivation to make a better impression, to improve, and to work more ambitiously toward the goals I had set for myself” (translated from German).

#### 4.2.2 Level 2—Learning (H1)

The knowledge test demonstrated acceptable internal consistency [Cronbach's α = 0.75, 95% CI (0.63, 0.85)]. On average, students answered a high proportion of items correctly at posttest [*M* = 0.87, *SD* = 0.09, 95% CI (0.84, 0.90)]. This mean proportion correct was significantly above the theoretical chance level of 0.50, *t* (40) = 27.31, *p* < 0.001, *d* = 4.26. As no pretest knowledge assessment was administered, this one-sample comparison against theoretical chance-level responding cannot be interpreted as evidence for intervention-induced learning gains; it establishes only that participants were not responding at random. Nevertheless, these results indicate a high level of topic-specific knowledge across the domains of growth mindset, grit, self-discipline, self-efficacy, and resilience, consistent with Hypothesis 1a.

Qualitative interview data converged with the quantitative findings and suggested that learning was not limited to factual knowledge. Participants reported a deeper understanding of grit, including the distinction between proximal and distal goals, and described reflecting on and revising prior beliefs about the malleability of abilities during adolescence. Several students also referred to the perceived usefulness of specific techniques, including mental contrasting with implementation intentions (Duckworth et al., 2011) and a more constructive approach to mistakes and feedback. For example, one student explained that she had previously attributed her difficulties in mathematics to genetic factors, but now believed that focused practice could lead to improvement, stating that although she had thought “it's genetic, because my mother can't do it,” she had come to see that “if you study and really concentrate, it can work, even for me” (translated from German). Similar reflections were expressed by other participants, who emphasized that “the brain can grow at any age” (translated from German) and that previously assumed fixed limitations (“if I can't do something, then I just can't do it”) were now perceived as changeable. Overall, these accounts indicate that participants reported learning outcomes that extended beyond correct responses on the knowledge test to more elaborated conceptual understandings and more malleable beliefs about learning and ability, consistent with Hypothesis 1b.

#### 4.2.3 Level 3—Behavior and training transfer after 3 months (RQ2)

At the 3-month follow-up (*n*_*FU*_ = 17), perceived training transfer was in the low-to-moderate range. Mean perceived application of the training contents in everyday life was *M* = 2.27 [*SD* = 0.78, 95% CI (1.87, 2.68)], while perceived usefulness of the trained non-cognitive skills was slightly higher [*M* = 2.68, *SD* = 0.89, 95% CI (2.23, 3.14); both measured on a 1–5 scale]. One-sample *t* tests against the neutral midpoint of 3 indicated that perceived application was significantly below the midpoint, *t* (16) = −3.82, *p* = 0.002, Cohen's *d* = −0.93. In contrast, perceived usefulness did not differ significantly from the midpoint, *t* (16) = −1.48, *p* = 0.159, *d* = −0.36.

Shapiro–Wilk tests on the post–follow-up difference scores indicated a significant departure from normality for perceived usefulness (*W* = 0.86, *p* = 0.016), while perceived application did not deviate significantly (*W* = 0.96, *p* = 0.611). Given the small follow-up sample (*n*_*FU*_ = 17) and the normality violation for perceived usefulness, longitudinal comparisons were conducted using Wilcoxon signed-rank tests as the primary inferential procedure, with parametric paired-samples *t* tests reported as a robustness check. Posttest values reported in this section refer to the *n*_*FU*_ = 17 participants who also provided follow-up data and therefore differ slightly from the full posttest sample reported in Section 4.2.1. Perceived application decreased substantially from posttest (*M* = 4.02, *SD* = 0.51) to follow-up (*M* = 2.27, *SD* = 0.78), corresponding to a mean change of −1.75 points, *V* = 0, *p* < 0.001, *d*_z_ = −2.42. Similarly, perceived usefulness declined from *M* = 3.91 (*SD* = 0.63) to *M* = 2.68 (*SD* = 0.89), yielding a mean change of −1.23 points, *V* = 0, *p* < 0.001, *d*_z_ = −1.67. The parametric tests produced consistent results. Because the posttest and follow-up items differed in framing (see Section 3.2.2), the magnitude of these changes cannot be attributed to declining transfer alone. These findings indicate a pronounced attenuation of initially high post-training evaluations over the 3-month period. Full statistics are reported in Supplementary Table 5.

Exploratory correlational analyses suggested moderate positive associations between post-intervention reactions and follow-up transfer indicators (Spearman's *r* ranging approximately from 0.44 to 0.71), indicating that participants with more favorable immediate post-training evaluations tended to report higher perceived transfer at follow-up.

No significant differences between participants with and without follow-up data emerged for post-training satisfaction, perceived application, perceived usefulness, or posttest knowledge scores (all |*t*s| ≤ 1.47, all *p* ≥ 0.15), with effect sizes ranging from negligible to moderate (Hedges' *g* = −0.03 to 0.44). Given that this comparison was itself based on small subgroups and that effect sizes reached *g* = 0.44, selective attrition cannot be ruled out. Full statistics are reported in Supplementary Table 6.

Qualitative interviews provided descriptive insight into how participants attempted to implement trained strategies in their everyday academic lives. Several students reported establishing structured learning routines (e.g., fixed study times, use of techniques such as the Pomodoro method), applying self-regulatory strategies (e.g., self-instruction, attentional refocusing during stressful phases, reduction of digital distractions), and engaging more actively with feedback. For example, one participant described using a fixed 1-hour study period after school to strengthen self-discipline, while another reported preparing for examinations by systematically completing multiple practice tests. Other accounts highlighted changes in attributional patterns, such as emphasizing effort and strategy use in challenging subjects, as well as concrete behavioral adjustments (e.g., uninstalling distracting applications or reframing critical feedback as informative rather than discouraging). Collectively, these qualitative accounts indicate that, despite overall declines in perceived transfer ratings, some participants continued to apply specific self-regulatory strategies and effort-related beliefs in their academic routines.

#### 4.2.4 Level 4a—Results: academic outcomes (H2)

Levene's tests indicated homogeneity of variances for both time points (Term1: *F* (1, 85) = 0.17, *p* = 0.683; Term2: *F* (1, 85) = 0.01, *p* = 0.913).

At baseline (Term 1), the experimental group exhibited slightly higher achievement scores than the control group (*M* = 10.6 vs. 10.1), although this difference was not statistically significant, *t* (81.0) = −1.38, *p* = 0.173, Hedges' *g* = −0.29.

Across both groups, mean academic achievement scores showed a modest increase from the first (Term 1) to the second term (Term 2). In the control group, mean grades increased from 10.12 [*SD* = 1.68, 95% CI (9.62, 10.62)] at Term 1 to 10.37 [*SD* = 1.88, 95% CI (9.81, 10.93)] at Term 2. Similarly, in the experimental group, mean grades rose from 10.64 [*SD* = 1.87, 95% CI (10.05, 11.23)] to 10.95 [*SD* = 1.97, 95% CI (10.33, 11.57)].

The mixed-design ANOVA revealed a significant main effect of time, *F* (1, 85) = 17.05, *p* < 0.001, η^2^_p_ = 0.167, indicating an overall improvement in academic achievement across both groups. This general improvement likely reflects a broader school-year trend rather than an intervention-specific effect. The main effect of group was not significant, *F* (1, 85) = 1.99, *p* = 0.162, η^2^_p_ = 0.02. The time × group interaction, which tests H2 directly, was non-significant, *F* (1, 85) = 0.12, *p* = 0.725, η^2^_p_ = 0.001.

The linear mixed-effects model corroborated these findings. The model showed a significant positive effect of time (estimate = 0.26, *p* = 0.007), whereas the time × group interaction was not significant (estimate = 0.05, 95% CI [−0.22, 0.31], *p* = 0.73).

Additional change-score analyses yielded converging results. The mean improvement from Term1 to Term2 was 0.26 points in the control group [95% CI (0.05, 0.46)] and 0.30 points in the experimental group [95% CI (0.13, 0.48)]. The difference between these change scores was not statistically significant, *t* (84.4) = −0.36, *p* = 0.72, with a negligible effect size (Hedges' *g* = −0.08). Full statistics are reported in Supplementary Table 7.

#### 4.2.5 Level 4b—Results: non-cognitive outcomes (H3)

Shapiro–Wilk tests on the pre–follow-up difference scores (*n*_*FU*_ = 17) indicated no significant departures from normality for any construct (all *W*s ≥ 0.95, all *p*s ≥ 0.513). Paired-samples *t* tests were therefore used as the primary inferential procedure. Descriptive statistics and test results are presented in Table 2.

**Table 2:** Pre–follow-up changes in non-cognitive target constructs (experimental group).

Construct	*n*	Pretest *M* (*SD*)	Follow-up *M* (*SD*)	Mean difference	95% CI of Difference	*t* (16)	*p*	*d_*z*_*
Growth mindset	17	3.39 (0.90)	3.88 (1.11)	0.49	(−0.10, 1.08)	1.76	0.098	0.43
Grit	17	3.29 (0.54)	3.36 (0.55)	0.07	(−0.10, 0.24)	0.89	0.384	0.22
Self-discipline	17	3.06 (0.42)	3.20 (0.53)	0.15	(−0.01, 0.30)	1.97	0.066	0.48
Self-efficacy	17	2.66 (0.41)	2.86 (0.52)	0.20	(0.00, 0.40)	2.12	0.050	0.51
Resilience	17	3.30 (0.67)	3.14 (0.92)	−0.17	(−0.52, 0.19)	−1.00	0.330	−0.24

Values are based on participants in the experimental group with complete follow-up data (*n*_*FU*_ = 17). Mean differences reflect follow-up minus pretest scores. Paired-samples *t* tests served as the primary inferential procedure; Shapiro–Wilk tests confirmed normality of all difference scores (all *W*s ≥ 0.95, all *p*s ≥ 0.513). *p* values shown in the table are uncorrected; none of the effects reached statistical significance after Holm correction for multiple comparisons. As a robustness check, Wilcoxon signed-rank tests yielded results consistent with the parametric analyses (all *p*_*Holm*_ ≥ 0.233). Effect sizes are reported as *d*_*z*_ (standardized mean change).

**Figure 1:**
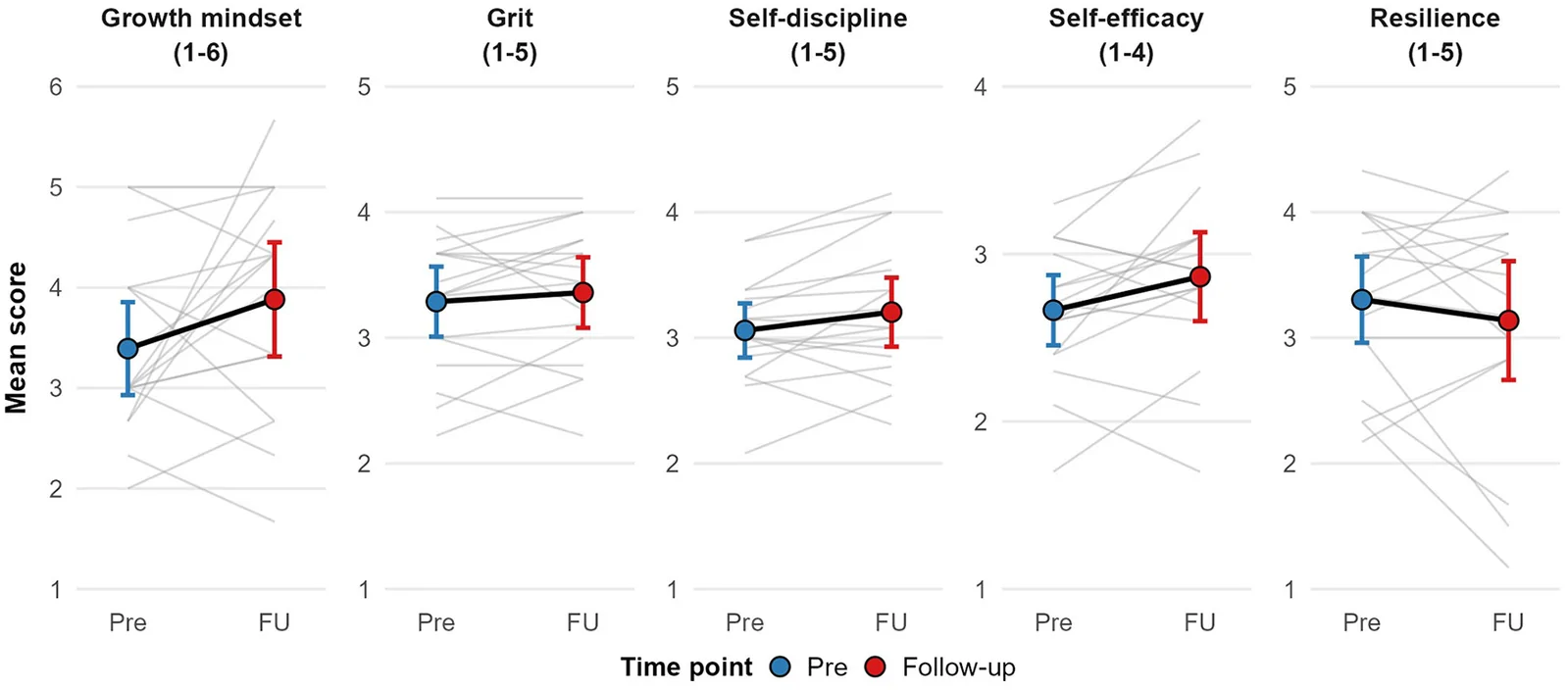
Pretest and 3-month follow-up scores on the five non-cognitive target constructs in the experimental group. Gray lines show individual trajectories of the *n*FU = 17 participants who provided complete pre–follow-up data; colored points show group means with 95% confidence intervals. Values correspond to Table 2. Each panel spans the full response range of the respective instrument (growth mindset 1–6; grit, self-discipline, and resilience 1–5; self-efficacy 1–4); scores are therefore not comparable across panels.

As shown in Table 2, mean scores increased from pretest to follow-up for growth mindset, grit, self-discipline, and self-efficacy, whereas resilience showed a small decrease. None of the observed changes reached conventional levels of statistical significance after adjustment for multiple comparisons using Holm's procedure (all corrected *p*s ≥ 0.250). Effect sizes ranged from small to moderate, with the largest effects observed for self-efficacy (*d*_z_ = 0.51) and self-discipline (*d*_z_ = 0.48), followed by growth mindset (*d*_z_ = 0.43). Changes in grit and resilience were small (|*d*_z_ | ≤ 0.24). As a robustness check, Wilcoxon signed-rank tests were conducted; results were consistent with the parametric analyses, with no construct reaching statistical significance after Holm correction (all corrected *p*s ≥ 0.233). The pattern of pretest-to-follow-up changes across all five constructs is illustrated in Figure 1. Full statistics are reported in Supplementary Table 8.

## 5 Discussion

The present study investigated the feasibility and preliminary effects of an integrated school-based training targeting five non-cognitive skills among German upper secondary school students. Using Kirkpatrick and Kirkpatrick (2006) four-level evaluation framework and a mixed-methods design, the study assessed training reactions, knowledge acquisition, behavioral transfer, academic outcomes, and changes in the targeted constructs over a 3-month follow-up period. Participants rated the training positively and showed high topic-specific knowledge at posttest (RQ1, H1); perceived transfer declined markedly over 3 months (RQ2); academic grades did not improve differentially between groups (H2); and changes in the targeted constructs were small to moderate but did not reach statistical significance after correction for multiple comparisons (H3).

Regarding RQ1 and H1, participants evaluated the training positively across all three reaction scales, and posttest knowledge was well above chance level. Although positive reactions are commonly observed at the lower levels of the Kirkpatrick framework (Kirkpatrick and Kirkpatrick, 2006), they are typically considered a necessary but not sufficient condition for behavioral change at higher evaluation levels. Qualitative interview data extended these findings by revealing that learning went beyond factual reproduction. Several participants reported revised attributional beliefs and described applying strategies such as goal decomposition and constructive failure interpretation, suggesting deeper conceptual processing consistent with mechanisms identified in wise psychological interventions (Walton and Wilson, 2018). Because no pretest knowledge assessment was administered, the knowledge test establishes that participants held a high level of topic-specific knowledge after the training, but it cannot show that this knowledge was acquired through it. The interview accounts speak more directly to change, as several participants explicitly contrasted their current understanding with beliefs they reported holding beforehand. Conclusions at Level 2 (Learning) therefore rest on self-reported conceptual change rather than on measured learning gains.

With respect to RQ2, the most noteworthy finding concerns the pronounced decline in perceived training transfer over the 3-month follow-up period. Yeager and Walton (2011) argued that the sustainability of brief psychological interventions depends critically on whether the surrounding context supports the targeted processes over time. In the present study, the training was implemented as a standalone program without booster sessions, teacher involvement, or curricular integration, providing limited contextual support for maintaining newly acquired skills. This absence of institutional reinforcement represents one plausible explanation for the observed transfer decline. A second explanation is methodological: the posttest items assessed anticipated applicability, whereas the follow-up items assessed retrospectively reported use (see Section 3.2.2). A shift from prospective expectation to retrospective report is well documented in training evaluation research, and the observed decline should therefore not be read as an estimate of fading transfer alone. However, the qualitative interview data revealed a different picture. The declines described above are based on the follow-up survey measures completed by the *n*_FU_ = 17 participants, whereas the interviews were conducted 1 month after the training with a subsample that overlaps with this group to an unknown degree (see Section 3.1). Within that subsample, individual participants described continued use of specific self-regulatory strategies such as structured study routines, attentional refocusing, and constructive feedback processing. This discrepancy between global self-report ratings and specific behavioral accounts suggests that standardized transfer measures may underestimate the degree to which discrete intervention components are selectively maintained.

Concerning H2, the absence of differential grade improvement between groups is consistent with meta-analytic evidence suggesting that the effects of non-cognitive interventions on academic achievement tend to be small. Sisk et al. (2018) reported only weak associations between growth mindset and academic performance, and Kraft (2020) noted that even intensive educational interventions rarely produce effects exceeding 0.10 to 0.15 standard deviations on objective academic outcomes. The modest dosage of six sessions, the distal nature of grades as an outcome variable, and the quasi-experimental design with self-selection likely contributed to the null finding.

Turning to H3, pretest-to-follow-up changes in the five target constructs revealed small to moderate effect sizes for self-efficacy, self-discipline, and growth mindset, none of which reached significance after Holm correction. Two explanations for this pattern are not mutually exclusive. The first is statistical: the follow-up sample was sensitive only to large within-person effects (*d*z ≥ 0.93), so the observed small-to-moderate effects fell below the detection threshold by design. The second follows from the distal-proximal continuum outlined in Section 2. The five constructs differ in how directly they are open to change within a single training day, and the observed pattern corresponds to these differences. The largest changes emerged for self-efficacy (*d*z = 0.51), conceptualized here as a set of future-oriented judgments about one's capability to cope with academic demands, responsive to mastery experiences and social persuasion (Bandura, 1977), both of which were incorporated into the training design, and for self-discipline (*d*z = 0.48), examined as a proximal capacity operating at the level of day-to-day behavioral regulation. Growth mindset (*d*z = 0.43) is the most distal of the five constructs, but it is also the one addressed most directly at the level of belief, and implicit theories of intelligence have been shown to shift in response to brief conceptual interventions (Blackwell et al., 2007; Yeager et al., 2019). The two constructs showing negligible change occupy different positions in this scheme. Grit reflects the consistency of interest and effort accumulated across extended periods and overlaps substantially with conscientiousness (Credé et al., 2017), which makes it the least plausible target for change over a 3-month interval following a single training day. Credé (2018) likewise found no support for the claim that grit is responsive to intervention, so the absence of change in this construct is consistent with prior evidence. Because grit and self-discipline correlated at r = 0.76 in this sample, the contrast between them should not be read as evidence that the training affected one construct and not the other. The self-discipline measure addresses day-to-day regulation, which the training exercises targeted directly, whereas the grit measure addresses perseverance sustained over far longer periods; the difference in observed change may therefore lie in what the two instruments capture rather than in the constructs themselves. Resilience, by contrast, was conceptualized here not as a stable trait but as a context-dependent process of adaptation to current demands (Kalisch et al., 2019; Rutter, 2011); scores on such a measure track concurrent circumstances, and the follow-up assessment fell within a period of ongoing academic demands. Taken together, these considerations suggest that the adequacy of a given training dosage is construct-specific rather than uniform: a single day may be sufficient to shift beliefs and proximal regulatory processes but insufficient to alter dispositions that consolidate over longer developmental timescales. This interpretation is *post hoc* and requires confirmation in adequately powered designs.

A recurrent theme across all four evaluation levels was that qualitative and quantitative data pointed to partially different conclusions about intervention effectiveness. At Level 2, interviews captured conceptual learning beyond what the knowledge test could measure; at Level 3, qualitative accounts revealed continued strategy use despite declining quantitative transfer ratings; and at Level 4, academic grades showed no differential improvement, yet participants described concrete changes in their approach to academic challenges. Standardized scales assess average change across broadly defined constructs, and distal outcomes such as grades reflect the cumulative influence of many factors beyond any single intervention. Neither fully captures the selective, context-dependent processes that brief interventions may set in motion. The qualitative component served to identify where and how such processes persisted at a level of specificity that standardized measures could not reach, illustrating the particular value of mixed-methods designs in underpowered pilot research.

## 6 Implications

The present findings suggest that integrated non-cognitive skills training is feasible within a regular school schedule and is perceived as relevant by upper secondary school students. Because the design included no comparison group for any psychological outcome and participants selected themselves into the training, the study does not permit conclusions about intervention effects. The qualitative data consistently indicated that participants acquired new conceptual understanding, revised their attributional beliefs, and continued to apply specific self-regulatory strategies beyond the training period. These insights would not have been captured through standardized quantitative measures alone, underscoring the importance of mixed-methods evaluation in pilot research.

These findings also carry implications for how non-cognitive skills interventions are evaluated. The divergence between standardized measures and qualitative accounts is consistent with evidence that the effects of non-cognitive interventions on distal academic outcomes tend to be small and often fall below conventional detection thresholds, even when more proximal psychological processes have been set in motion. Future evaluations of brief school-based programs may benefit from supplementing standardized instruments with structured qualitative protocols and proximal behavioral indicators to capture a fuller picture of intervention effects, particularly in pilot studies where small samples limit statistical power.

Rather than treating non-cognitive skills training as a standalone supplement, the present results call for embedding intervention principles into everyday instructional practice. Educators may benefit from integrating growth-oriented elements into regular instruction, for instance, by designing iterative assignments that normalize mistakes and emphasize revision, thereby reinforcing the belief that abilities are developable (Blackwell et al., 2007; Dweck, 2006). The central role of self-efficacy observed in this study further supports structuring learning activities around early mastery experiences, as research consistently indicates that confidence built through graduated success fosters sustained engagement and self-regulation (Schunk, 1981; Zimmerman, 2000). Approaches focusing on feedback, reflection, and metacognition have been identified as among the most effective non-cognitive educational strategies (Stankov and Lee, 2014) and could serve as practical vehicles for sustaining the processes initiated by the training.

Regarding academic outcomes, the null finding for grades is consistent with evidence that changes in non-cognitive skills translate into measurable achievement gains primarily when the broader learning environment actively supports these processes (Yeager et al., 2016b). Because modifying institutional structures is resource-intensive and often beyond the scope of individual intervention studies, future research should examine whether such training programs improve more proximal psychological outcomes (such as academic motivation, optimism, or learning-related self-regulation) that are more directly targeted by the intervention content. Improvements in these constructs may, in turn, exert moderating or mediating effects on more distal outcomes such as grades, offering a more sensitive and theoretically grounded evaluation approach.

Finally, the Kirkpatrick framework proved useful for structuring the multi-level evaluation of this educational intervention, as it enabled a differentiated assessment of outcomes ranging from participant satisfaction to behavioral transfer and academic performance. Its application in school-based research may benefit from greater attention to the conditions under which higher-level outcomes (particularly at Levels 3 and 4) can realistically be expected given the intervention dosage, contextual constraints, and the degree of institutional support available.

## 7 Limitations and future directions

Beyond the statistical power constraints and self-selection issues discussed above, several additional limitations should be noted. The sample was drawn from a single German upper secondary school, limiting generalizability. With *n*EG = 41 at posttest and *n*FU = 17 at follow-up, the study can describe the direction and approximate magnitude of change but cannot establish it with precision, and none of the estimates reported here should be treated as population values. No baseline questionnaire data were collected for the control group, precluding direct comparison on the target constructs at pretest. Because the control group contributed grade data only, every psychological outcome in this study is based on within-group change in a self-selected sample and is therefore open to maturation, seasonal variation in academic demands, regression to the mean, and demand characteristics. Intervention effects and selection effects cannot be separated in this design. Only 17 of the 41 participants (41.5%) provided follow-up data, so all pretest-to-follow-up analyses rest on a small subsample. The attrition analysis reported in Section 4.2.3 detected no systematic differences, but it is itself insensitive to small and moderate effects. Part of the shortfall is attributable to the administration setting: pretest and posttest were completed on site under supervision, whereas the follow-up battery was completed remotely and unsupervised 3 months later. Unsupervised administration is commonly associated with lower response rates, which offers a non-selective explanation alongside the possibility of differential dropout. All non-cognitive constructs were assessed via self-report, implementation fidelity was not formally assessed, and the absence of an active control condition leaves open the possibility that demand characteristics contributed to observed differences. Regarding the qualitative component, initial segment-level intercoder agreement was moderate (κ = 0.30), although document-level agreement was substantially higher (76%) and all discrepancies were resolved through consensus coding.

Future research should address these limitations through a cluster-randomized design with multiple schools and an active control condition. The strong intercorrelation between grit and self-discipline observed here (*r* = 0.76) aligns with critiques of grit's discriminant validity and suggests that future iterations of the training may combine these constructs into a single self-regulation module. Booster sessions or teacher-led reinforcement may help sustain transfer effects (Yeager and Walton, 2011). Combining standardized self-reports with behavioral observation, experience sampling, or structured qualitative protocols would allow future studies to distinguish where intervention effects manifest from where they remain below the detection threshold of conventional measures. Examining potential moderators would further help clarify for whom and under what conditions such programs produce the most benefit (Yeager et al., 2019).

## Data Availability

The raw data supporting the conclusions of this article will be made available by the authors, without undue reservation.

## References

[B1] Alhadabi A. Karpinski A. C. (2020). Grit, self-efficacy, achievement orientation goals, and academic performance in university students. Int. J. Adolesc. Youth 25, 519–535. doi: 10.1080/02673843.2019.1679202

[B2] Alva S. A. (1991). Academic invulnerability among Mexican-American students: the importance of protective resources and appraisals. His. J. Behav. Sci. 13, 18–34. doi: 10.1177/07399863910131002

[B3] Anwar Z. Hanurawan F. Chusniyah T. Setiyowati N. Rehman S. (2024). Measurement models and predictors of student academic success: a systematic literature review. Sci.: Soc. Sci. Human. 3, 29–42. doi: 10.51773/sssh.v3i1.252

[B4] Ayala J. C. Manzano G. (2018). Academic performance of first-year university students: the influence of resilience and engagement. High. Educ. Res. Dev. 37, 1321–1335. doi: 10.1080/07294360.2018.1502258

[B5] Bandura A. (1977). Self-efficacy: toward a unifying theory of behavioral change. Psychol. Rev. 84, 191–215. doi: 10.1037/0033-295X.84.2.191 847061

[B6] Bandura A. (1995). Self-Efficacy in Changing Societies. Cambridge: Cambridge University Press. doi: 10.1017/CBO9780511527692

[B7] Bandura A. Locke E. A. (2003). Negative self-efficacy and goal effects revisited. J. Appl. Psychol. 88, 87–99. doi: 10.1037/0021-9010.88.1.87 12675397

[B8] Bandura A. Schunk D. H. (1981). Cultivating competence, self-efficacy, and intrinsic interest through proximal self-motivation. J. Pers. Soc. Psychol. 41, 586–598. doi: 10.1037/0022-3514.41.3.586

[B9] Baumeister R. F. Exline J. J. (2000). Self-control, morality, and human strength. J. Soc. Clin. Psychol. 19, 29–42. doi: 10.1521/jscp.2000.19.1.29

[B10] Bertrams A. Dickhäuser O. (2009). Messung dispositioneller Selbstkontroll-Kapazität. Diagnostica 55, 2–10. doi: 10.1026/0012-1924.55.1.2

[B11] Bishara A. J. Hittner J. B. (2012). Testing the significance of a correlation with nonnormal data: comparison of Pearson, Spearman, transformation, and resampling approaches. Psychol. Methods 17, 399–417. doi: 10.1037/a0028087 22563845

[B12] Blackwell L. S. Trzesniewski K. H. Dweck C. S. (2007). Implicit theories of intelligence predict achievement across an adolescent transition: a longitudinal study and an intervention. Child Dev. 78, 246–263. doi: 10.1111/j.1467-8624.2007.00995.x 17328703

[B13] Braun V. Clarke V. (2006). Using thematic analysis in psychology. Qual. Res. Psychol. 3, 77–101. doi: 10.1191/1478088706qp063oa

[B14] Brennan R. L. Prediger D. J. (1981). Coefficient kappa: some uses, misuses, and alternatives. Educ. Psychol. Meas. 41, 687–699. doi: 10.1177/001316448104100307

[B15] Brewer M. L. van Kessel G. Sanderson B. Naumann F. Lane M. Reubenson A. . (2019). Resilience in higher education students: a scoping review. High. Educ. Res. Dev. 38, 1105–1120. doi: 10.1080/07294360.2019.1626810

[B16] Breyer B. Danner D. (2015). Grit Scale for Perseverance and Passion for Long-Term Goals. Mannheim: ZIS - Zusammenstellung sozialwissenschaftlicher Items und Skalen, published by GESIS - Leibniz Institute for the Social Sciences.

[B17] Burgoyne A. P. Hambrick D. Z. Moser J. S. Burt S. A. (2018). Analysis of a mindset intervention. J. Res. Pers. 77, 21–30. doi: 10.1016/j.jrp.2018.09.004

[B18] Buzzetto-Hollywood N. Mitchell B. Hill A. (2019). Introducing a mindset intervention to improve student success. Interdiscip. J. E-Skills Lifelong Learn. 15, 135–155. doi: 10.28945/4465

[B19] Chmitorz A. Wenzel M. Stieglitz R.-D. Kunzler A. Bagusat C. Helmreich I. . (2018). Population-based validation of a German version of the brief resilience scale. PLoS ONE 13, e0192761. doi: 10.1371/journal.pone.0192761 29438435 PMC5811014

[B20] Claro S. Paunesku D. Dweck C. S. (2016). Growth mindset tempers the effects of poverty on academic achievement. Proc. Natl. Acad. Sci. U. S. A. 113, 8664–8668. doi: 10.1073/pnas.1608207113 27432947 PMC4978255

[B21] Credé M. (2018). What shall we do about grit? A critical review of what we know and what we don't know. Educ. Res. 47, 606–611. doi: 10.3102/0013189X18801322

[B22] Credé M. Tynan M. C. Harms P. D. (2017). Much ado about grit: a meta-analytic synthesis of the grit literature. J. Pers. Soc. Psychol. 113, 492–511. doi: 10.1037/pspp0000102 27845531

[B23] Creswell J. W. Plano Clark V. L. (2018). Designing and Conducting Mixed Methods Research (3rd edn.). Los Angeles, CA: Sage.

[B24] Curran P. J. West S. G. Finch J. F. (1996). The robustness of test statistics to nonnormality and specification error in confirmatory factor analysis. Psychol. Methods 1, 16–29. doi: 10.1037/1082-989X.1.1.16

[B25] DeBacker T. K. Heddy B. C. Kershen J. L. Crowson H. M. Looney K. Goldman J. A. (2018). Effects of a one-shot growth mindset intervention on beliefs about intelligence and achievement goals. Educ. Psychol. 38, 711–733. doi: 10.1080/01443410.2018.1426833

[B26] Degol J. L. Wang M.-T. Zhang Y. Allerton J. (2018). Do growth mindsets in math benefit females? Identifying pathways between gender, mindset, and motivation. J. Youth Adolesc. 47, 976–990. doi: 10.1007/s10964-017-0739-8 28889203

[B27] Denovan A. Macaskill A. (2017). Stress and subjective well-being among first year UK undergraduate students. J. Happiness Stud. 18, 505–525. doi: 10.1007/s10902-016-9736-y

[B28] Duckworth A. (2009). Self-discipline is empowering. Phi Delta Kappan 90, 536. doi: 10.1177/003172170909000720

[B29] Duckworth A. Grant H. Loew B. Oettingen G. Gollwitzer P. M. (2011). Self-regulation strategies improve self-discipline in adolescents: benefits of mental contrasting and implementation intentions. Educ. Psychol. 31, 17–26. doi: 10.1080/01443410.2010.506003

[B30] Duckworth A. Gross J. J. (2014). Self-control and grit: related but separable determinants of success. Curr. Dir. Psychol. Sci. 23, 319–325. doi: 10.1177/0963721414541462 26855479 PMC4737958

[B31] Duckworth A. Peterson C. Matthews M. D. Kelly D. R. (2007). Grit: perseverance and passion for long-term goals. J. Pers. Soc. Psychol. 92, 1087–1101. doi: 10.1037/0022-3514.92.6.1087 17547490

[B32] Duckworth A. Seligman M. E. P. (2005). Self-discipline outdoes IQ in predicting academic performance of adolescents. Psychol. Sci. 16, 939–944. doi: 10.1111/j.1467-9280.2005.01641.x 16313657

[B33] Durlak J. A. Weissberg R. P. Dymnicki A. B. Taylor R. D. Schellinger K. B. (2011). The impact of enhancing students' social and emotional learning: a meta-analysis of school-based universal interventions. Child Dev. 82, 405–432. doi: 10.1111/j.1467-8624.2010.01564.x 21291449

[B34] Dweck C. S. (1999). Self-Theories: Their Role in Motivation, Personality, and Development. Philadelphia, PA: Psychology Press.

[B35] Dweck C. S. (2006). Mindset: The New Psychology of Success (1st edn.). New York, NY: Random House.

[B36] Dweck C. S. (2017). Mindset: Changing the Way You Think to Fulfil Your Potential (Updated edn.). London: Robinson.

[B37] Dweck C. S. (2018). Growth mindset interventions yield impressive results. The Conversation. Available at: https://theconversation.com/growth-mindset-interventions-yield-impressive-results-97423 (Accessed July 22, 2025).

[B38] Dweck C. S. Chiu C. Hong Y. (1995). Implicit theories and their role in judgments and reactions: a word from two perspectives. Psychol. Inq. 6, 267–285. doi: 10.1207/s15327965pli0604_1

[B39] Dweck C. S. Leggett E. L. (1988). A social-cognitive approach to motivation and personality. Psychol. Rev. 95, 256–273. doi: 10.1037/0033-295X.95.2.256

[B40] Eccles J. (2009). Who am I and what am I going to do with my life? Personal and collective identities as motivators of action. Educ. Psychol. 44, 78–89. doi: 10.1080/00461520902832368

[B41] Eskreis-Winkler L. Shulman E. P. Beal S. A. Duckworth A. L. (2014). The grit effect: predicting retention in the military, the workplace, school and marriage. Front. Psychol. 5:36. doi: 10.3389/fpsyg.2014.00036 24550863 PMC3910317

[B42] Faul F. Erdfelder E. Lang A.-G. Buchner A. (2007). G^*^Power 3: a flexible statistical power analysis program for the social, behavioral, and biomedical sciences. Behav. Res. Methods 39, 175–191. doi: 10.3758/BF03193146 17695343

[B43] Feldt L. S. (1965). The approximate sampling distribution of Kuder-Richardson reliability coefficient twenty. Psychometrika 30, 357–370. doi: 10.1007/BF02289499 5216223

[B44] Fredricks J. A. Blumenfeld P. C. Paris A. H. (2004). School engagement: potential of the concept, state of the evidence. Rev. Educ. Res. 74, 59–109. doi: 10.3102/00346543074001059

[B45] Funder D. C. Block J. H. Block J. (1983). Delay of gratification: some longitudinal personality correlates. J. Pers. Soc. Psychol. 44, 1198–1213. doi: 10.1037/0022-3514.44.6.1198

[B46] Furlong M. J. You S. Renshaw T. L. Smith D. C. O'Malley M. D. (2014). Preliminary development and validation of the social and emotional health survey for secondary school students. Soc. Indic. Res. 117, 1011–1032. doi: 10.1007/s11205-013-0373-0

[B47] Gist M. E. Mitchell T. R. (1992). Self-efficacy: a theoretical analysis of its determinants and malleability. Acad. Manag. Rev. 17, 183–211. doi: 10.2307/258770

[B48] Hagger M. S. Hamilton K. (2019). Grit and self-discipline as predictors of effort and academic attainment. Br. J. Educ. Psychol. 89, 324–342. doi: 10.1111/bjep.12241 30101970

[B49] Heckman J. J. Kautz T. (2012). Hard evidence on soft skills. Labour Econ. 19, 451–464. doi: 10.1016/j.labeco.2012.05.014 23559694 PMC3612993

[B50] Heckman J. J. Rubinstein Y. (2001). The importance of noncognitive skills: lessons from the GED Testing Program. American Economic Review 91, 145–149. doi: 10.1257/aer.91.2.145

[B51] Hinz A. Schumacher J. Albani C. Schmid G. Brähler E. (2006). Bevölkerungsrepräsentative Normierung der Skala zur Allgemeinen Selbstwirksamkeitserwartung. Diagnostica 52, 26–32. doi: 10.1026/0012-1924.52.1.26

[B52] Holm S. (1979). A simple sequentially rejective multiple test procedure. Scand. J. Stat. 6, 65–70. doi: 10.2307/4615733

[B53] Hwang M. H. Lim H. J. Ha H. S. (2018). Effects of grit on the academic success of adult female students at Korean open university. Psychol. Rep. 121, 705–725. doi: 10.1177/0033294117734834 29298549

[B54] Joyce S. Shand F. Tighe J. Laurent S. J. Bryant R. A. Harvey S. B. (2018). Road to resilience: a systematic review and meta-analysis of resilience training programmes and interventions. BMJ Open 8:e017858. doi: 10.1136/bmjopen-2017-017858 29903782 PMC6009510

[B55] Kalisch R. Cramer A. O. J. Binder H. Fritz J. Leertouwer I. Lunansky G. . (2019). Deconstructing and reconstructing resilience: a dynamic network approach. Perspect. Psychol. Sci. 14, 765–777. doi: 10.1177/1745691619855637 31365841

[B56] Kamins M. L. Dweck C. S. (1999). Person versus process praise and criticism: implications for contingent self-worth and coping. Dev. Psychol. 35, 835–847. doi: 10.1037/0012-1649.35.3.835 10380873

[B57] Kirkpatrick D. L. Kirkpatrick J. D. (2006). Evaluating Training Programs: The Four Levels (3rd edn.). San Francisco, CA: Berrett-Koehler.

[B58] Kraft M. A. (2020). Interpreting effect sizes of education interventions. Educ. Res. 49, 241–253. doi: 10.3102/0013189X20912798

[B59] Kuncel N. R. Hezlett S. A. (2010). Fact and fiction in cognitive ability testing for admissions and hiring decisions. Curr. Dir. Psychol. Sci. 19, 339–345. doi: 10.1177/0963721410389459

[B60] Landis J. R. Koch G. G. (1977). The measurement of observer agreement for categorical data. Biometrics 33, 159. doi: 10.2307/2529310 843571

[B61] Li J. Ma T. Lee C. S. (2025). Grit as a moderator: the mediating role of self-efficacy and academic performance between conscientiousness and life satisfaction among college students. Front. Psychol. 16:1665636. doi: 10.3389/fpsyg.2025.1665636 41341724 PMC12669109

[B62] Luthar S. S. Cicchetti D. Becker B. (2000). The construct of resilience: a critical evaluation and guidelines for future work. Child Dev. 71, 543–562. doi: 10.1111/1467-8624.00164 10953923 PMC1885202

[B63] Macnamara B. N. Burgoyne A. P. (2023). Do growth mindset interventions impact students' academic achievement? A systematic review and meta-analysis with recommendations for best practices. Psychol. Bull. 149, 133–173. doi: 10.1037/bul0000352 36326645

[B64] Mahatmaharti A. Nali Brata D. Nizarudin Wajdi M. (2019). Cognitive modeling effect: enhancing student self-discipline through self-instructional training. Int. J. Mech. Eng. Technol. 10, 818–827. Available online at: https://ssrn.com/abstract=3452409

[B65] Malanchini M. Allegrini A. G. Nivard M. G. Biroli P. Rimfeld K. Cheesman R. . (2024). Genetic associations between non-cognitive skills and academic achievement over development. Nat. Human Behav. 8, 2034–2046. doi: 10.1038/s41562-024-01967-9 39187715 PMC11493678

[B66] Martin A. J. (2002). Motivation and academic resilience: developing a model for student enhancement. Aust. J. Educ. 46, 34–49. doi: 10.1177/000494410204600104

[B67] Martin A. J. Marsh H. W. (2006). Academic resilience and its psychological and educational correlates: a construct validity approach. Psychol. Sch. 43, 267–281. doi: 10.1002/pits.20149

[B68] Mirza T. I. Yasmeen R. Mahboob U. (2021). Nurturing grit among medical students. Pak. J. Med. Sci. 37, 531–535. doi: 10.12669/pjms.37.2.2999 33679945 PMC7931310

[B69] Mischel W. Shoda Y. Rodriguez M. I. (1989). Delay of gratification in children. Science 244, 933–938. doi: 10.1126/science.2658056 2658056

[B70] Multon K. D. Brown S. D. Lent R. W. (1991). Relation of self-efficacy beliefs to academic outcomes: a meta-analytic investigation. J. Couns. Psychol. 38, 30–38. doi: 10.1037/0022-0167.38.1.30

[B71] Ning H. K. Downing K. (2010). The reciprocal relationship between motivation and self-regulation: a longitudinal study on academic performance. Learn. Individ. Differ. 20, 682–686. doi: 10.1016/j.lindif.2010.09.010

[B72] Nisbett R. E. Aronson J. Blair C. Dickens W. Flynn J. Halpern D. F. . (2012). Intelligence: new findings and theoretical developments. Am. Psychol. 67, 130–159. doi: 10.1037/a0026699 22233090

[B73] Pajares F. Kranzler J. (1995). Self-efficacy beliefs and general mental ability in mathematical problem-solving. Contemp. Educ. Psychol. 20, 426–443. doi: 10.1006/ceps.1995.1029

[B74] Paunesku D. Walton G. M. Romero C. Smith E. N. Yeager D. S. Dweck C. S. (2015). Mind-set interventions are a scalable treatment for academic underachievement. Psychol. Sci. 26, 784–793. doi: 10.1177/0956797615571017 25862544

[B75] Perkins-Gough D. (2013). The significance of grit: a conversation with Angela Lee Duckworth. Educ. Leadersh. 71, 14–20.

[B76] Plomin R. Deary I. J. (2015). Genetics and intelligence differences: five special findings. Mol. Psychiatry 20, 98–108. doi: 10.1038/mp.2014.105 25224258 PMC4270739

[B77] Polirstok S. (2017). Strategies to improve academic achievement in secondary school students: perspectives on grit and mindset. SAGE Open 7. doi: 10.1177/2158244017745111. [Epub ahead of print].

[B78] R Core Team (2026). R: A Language and Environment for Statistical Computing. Vienna: R Foundation for Statistical Computing. Available online at: https://www.R-project.org/

[B79] Rammstedt B. Grüning D. J. Lechner C. M. (2024). Measuring growth mindset: validation of a three-item and a single-item scale in adolescents and adults. Eur. J. Psychol. Assess. 40, 84–95. doi: 10.1027/1015-5759/a000735

[B80] Roberts B. W. Lejuez C. Krueger R. F. Richards J. M. Hill P. L. (2014). What is conscientiousness and how can it be assessed? Dev. Psychol. 50, 1315–1330. doi: 10.1037/a0031109 23276130

[B81] Romer D. Duckworth A. L. Sznitman S. Park S. (2010). Can adolescents learn self-control? Delay of gratification in the development of control over risk taking. Prev. Sci. 11, 319–330. doi: 10.1007/s11121-010-0171-8 20306298 PMC2964271

[B82] Rosen J. A. Glennie E. J. Dalton B. W. Lennon J. M. Bozick R. N. (2010). Noncognitive Skills in the Classroom: New Perspectives on Educational Research. Research Triangle Park, NC: RTI Press. doi: 10.3768/rtipress.2010.bk.0004.1009

[B83] Rutter M. (2011). “Resilience reconsidered: conceptual considerations, empirical findings, and policy implications,” in Handbook of Early Childhood Intervention, eds. J. P. Shonkoff, S. J. Meisels, and E. F. Zigler (Cambridge: Cambridge University Press), 651–682. doi: 10.1017/CBO9780511529320.030

[B84] Santos I. Petroska-Beska V. Carneiro P. Eskreis-Winkler L. Boudet A. M. M. Berniell I. . (2022). Can grit be taught? Lessons from a nationwide field experiment with middle-school students. IZA Discussion Paper No. 15588 [Preprint]. Available at: https://ssrn.com/abstract=4233803 (Accessed June, 11 2025).

[B85] Schmidt F. T. C. (2021). Beharrlichkeit und beständiges Interesse – die Bedeutung von Grit für den Bildungserfolg. Rep. Psychol. 46, 12–15.

[B86] Schunk D. H. (1981). Modeling and attributional effects on children's achievement: a self-efficacy analysis. J. Educ. Psychol. 73, 93–105. doi: 10.1037/0022-0663.73.1.93

[B87] Schwarzer R. Jerusalem M. (2003). SWE – Skala zur allgemeinen Selbstwirksamkeitserwartung. Open Test Archive. Leibniz-Institut für Psychologie (ZPID). doi: 10.23668/PSYCHARCHIVES.4515

[B88] Sisk V. F. Burgoyne A. P. Sun J. Butler J. L. Macnamara B. N. (2018). To what extent and under which circumstances are growth mind-sets important to academic achievement? Two meta-analyses. Psychol. Sci. 29, 549–571. doi: 10.1177/0956797617739704 29505339

[B89] Smith B. W. Dalen J. Wiggins K. Tooley E. Christopher P. Bernard J. (2008). The brief resilience scale: assessing the ability to bounce back. Int. J. Behav. Med. 15, 194–200. doi: 10.1080/10705500802222972 18696313

[B90] Smithers L. G. Sawyer A. C. P. Chittleborough C. R. Davies N. M. Davey Smith G. Lynch J. W. (2018). A systematic review and meta-analysis of effects of early life non-cognitive skills on academic, psychosocial, cognitive and health outcomes. Nat. Human Behav. 2, 867–880. doi: 10.1038/s41562-018-0461-x 30525112 PMC6277013

[B91] Soruç A. Pawlak M. Yuksel D. Horzum B. (2022). Investigating the impact of linguistic and non-linguistic factors on EMI academic success. System 107, 102794. doi: 10.1016/j.system.2022.102794

[B92] Stankov L. Lee J. (2014). Quest for the best non-cognitive predictor of academic achievement. Educ. Psychol. 34, 1–8. doi: 10.1080/01443410.2013.858908

[B93] Steinmayr R. Weidinger A. F. Schwinger M. Spinath B. (2019). The importance of students' motivation for their academic achievement – replicating and extending previous findings. Front. Psychol. 10:1730. doi: 10.3389/fpsyg.2019.01730 31417459 PMC6685139

[B94] Thomas L. J. Asselin M. (2018). Promoting resilience among nursing students in clinical education. Nurse Educ. Pract. 28, 231–234. doi: 10.1016/j.nepr.2017.10.001 29128734

[B95] Usher E. L. Pajares F. (2008). Self-efficacy for self-regulated learning. A validation study. Educ. Psychol. Meas. 68, 443–463. doi: 10.1177/0013164407308475

[B96] VERBI Software (2026). MAXQDA Analytics Pro (Release 26.2.1) [Computer software]. Berlin: VERBI Software. Consult. Sozialforschung GmbH. Available online at: https://www.maxqda.com

[B97] Vuong M. Brown-Welty S. Tracz S. (2010). The effects of self-efficacy on academic success of first-generation college sophomore students. J. Coll. Stud. Dev. 51, 50–64. doi: 10.1353/csd.0.0109

[B98] Walton G. M. Wilson T. D. (2018). Wise interventions: psychological remedies for social and personal problems. Psychol. Rev. 125, 617–655. doi: 10.1037/rev0000115 30299141

[B99] Wang M. C. Gordon E. W. (Eds.). (1994). Educational resilience in inner-city America: challenges and prospects. Hillsdale, NJ: Erlbaum.

[B100] Watts T. W. Duncan G. J. Quan H. (2018). Revisiting the Marshmallow test: a conceptual replication investigating links between early delay of gratification and later outcomes. Psychol. Sci. 29, 1159–1177. doi: 10.1177/0956797618761661 29799765 PMC6050075

[B101] Wolters C. A. Hussain M. (2015). Investigating grit and its relations with college students' self-regulated learning and academic achievement. Metacogn. Learn. 10, 293–311. doi: 10.1007/s11409-014-9128-9

[B102] Yeager D. S. Carroll J. M. Buontempo J. Cimpian A. Woody S. Crosnoe R. . (2022). Teacher mindsets help explain where a growth-mindset intervention does and doesn't work. Psychol. Sci. 33, 18–32. doi: 10.1177/09567976211028984 34936529 PMC8985222

[B103] Yeager D. S. Hanselman P. Walton G. M. Murray J. S. Crosnoe R. Muller C. . (2019). A national experiment reveals where a growth mindset improves achievement. Nature 573, 364–369. doi: 10.1038/s41586-019-1466-y 31391586 PMC6786290

[B104] Yeager D. S. Romero C. Paunesku D. Hulleman C. S. Schneider B. Hinojosa C. . (2016a). Using design thinking to improve psychological interventions: the case of the growth mindset during the transition to high school. J. Educ. Psychol. 108, 374–391. doi: 10.1037/edu0000098 27524832 PMC4981081

[B105] Yeager D. S. Walton G. M. (2011). Social-psychological interventions in education. They're not magic. Rev. Educ. Res. 81, 267–301. doi: 10.3102/0034654311405999

[B106] Yeager D. S. Walton G. M. Brady S. T. Akcinar E. N. Paunesku D. Keane L. . (2016b). Teaching a lay theory before college narrows achievement gaps at scale. Proc. Natl. Acad. Sci. U. S. A. 113, E3341–E3348. doi: 10.1073/pnas.1524360113 27247409 PMC4914175

[B107] York T. Gibson C. Rankin S. (2015). Defining and measuring academic success. Pract. Assess. Res. Eval. 20, 1–20. doi: 10.7275/hz5x-tx03

[B108] Zhao F. Meng W. Li F. Zhou L. (2021). Overview and insight from the China research report on the global youth survey of social and emotional skills by the organization for economic cooperation and development. Best Evid. Chin. Educ. 9, 1169–1195. doi: 10.15354/bece.21.re055

[B109] Zimmerman B. J. (2000). Self-efficacy: an essential motive to learn. Contemp. Educ. Psychol. 25, 82–91. doi: 10.1006/ceps.1999.1016 10620383

[B110] Zimmerman B. J. Kitsantas A. (2002). Acquiring writing revision and self-regulatory skill through observation and emulation. J. Educ. Psychol. 94, 660–668. doi: 10.1037/0022-0663.94.4.660

