# Supplementary material for: Beyond standardized measures: a mixed-methods pilot study of integrated non-cognitive skills training in German upper secondary students
Source: Front Psychol. 2026 Aug 26;17:1918459. doi: 10.3389/fpsyg.2026.1918459 (PMC13553911; doi:10.3389/fpsyg.2026.1918459)
Supplement: Supplementary file 1 [file Data_Sheet_1.docx]

**Supplementary Material**

**Supplementary Table 1.** *Level 1 (Reaction): Posttest Evaluation Items*

| **No.** | **German** | **English** | **Scale** |
| --- | --- | --- | --- |
| SAT_01 | Wie bewerten Sie den Inhalt des Trainings? | How do you rate the content of the training? | Satisfaction |
| SAT_02 | Wie gut war die Trainerin vorbereitet? | How well was the trainer prepared? | Satisfaction |
| SAT_03 | Wie gut war die Balance zwischen theoretischen Inhalten und praktischen Übungen? | How good was the balance between theoretical content and practical exercises? | Satisfaction |
| SAT_04 | Wie fanden Sie die Methoden und Aktivitäten im Training? (z. B. Gruppenarbeiten, Diskussionen, Übungen) | How did you find the methods and activities in the training? (e.g., group work, discussions, exercises) | Satisfaction |
| SAT_05 | Wie beurteilen Sie die Organisation und den Ablauf des Trainings? (z. B. Pausen, Zeitplan, Materialien) | How do you rate the organization and structure of the training? (e.g., breaks, schedule, materials) | Satisfaction |
| APP_01 (R) | Glauben Sie, dass das Gelernte Ihnen helfen wird, Ihre schulischen Leistungen zu verbessern? | Do you believe that what you learned will help you improve your academic performance? | Application |
| APP_02 | War das Training auf Ihre aktuellen Herausforderungen in der Schule anwendbar? | Was the training applicable to your current challenges at school? | Application |
| APP_03 | Hat Sie das Training motiviert, Ihre Fähigkeiten im Alltag zu verbessern? | Did the training motivate you to improve your skills in everyday life? | Application |
| USE_01 | Wie hilfreich fanden Sie das Thema „Growth Mindset" für Ihre persönliche Entwicklung und Ihre Schulleistungen? | How helpful did you find the topic "growth mindset" for your personal development and academic performance? | Usefulness |
| USE_02 | Wie hilfreich fanden Sie das Thema „Grit" für Ihre persönliche Entwicklung und Ihre Schulleistungen? | How helpful did you find the topic "grit" for your personal development and academic performance? | Usefulness |
| USE_03 | Wie hilfreich fanden Sie das Thema „Selbstdisziplin" für Ihre persönliche Entwicklung und Ihre Schulleistungen? | How helpful did you find the topic "self-discipline" for your personal development and academic performance? | Usefulness |
| USE_04 | Wie hilfreich fanden Sie das Thema „Selbstwirksamkeit" für Ihre persönliche Entwicklung und Ihre Schulleistungen? | How helpful did you find the topic "self-efficacy" for your personal development and academic performance? | Usefulness |
| USE_05 | Wie hilfreich fanden Sie das Thema „Resilienz" für Ihre persönliche Entwicklung und Ihre Schulleistungen? | How helpful did you find the topic "resilience" for your personal development and academic performance? | Usefulness |

*Note.* All items were rated on a 5-point Likert-type scale (1 = not at all to 5 = very much). (R) = reverse-coded. Internal consistency in the present sample: α_SAT_ = .73, α_APP_ = .71, α_USE_ = .84. Corresponds to Section 3.2.2 of the main text.

**Supplementary Table 2.** *Level 3 (Behavior): Follow-Up Evaluation Items*

| **No.** | **German** | **English** | **Scale** |
| --- | --- | --- | --- |
| FU_APP_01 | Konnten Sie die Inhalte des Trainings in den letzten drei Monaten auf Ihre Herausforderungen in der Schule anwenden? | Were you able to apply the training contents to your challenges at school over the past three months? | FU Application |
| FU_APP_02 | Wie hilfreich waren die Methoden und Aktivitäten aus dem Training (z. B. Gruppenarbeiten, Diskussionen, Übungen) für Ihren Alltag in den letzten drei Monaten? | How helpful were the methods and activities from the training (e.g., group work, discussions, exercises) for your everyday life over the past three months? | FU Application |
| FU_APP_03 | Hat das Training Sie in den letzten drei Monaten motiviert, Ihre Fähigkeiten im Alltag zu verbessern? | Did the training motivate you to improve your skills in everyday life over the past three months? | FU Application |
| FU_USE_01 | Wie hilfreich war das Thema „Growth Mindset" in den letzten drei Monaten für Ihre persönliche Entwicklung und Ihre Schulleistungen? | How helpful was the topic "growth mindset" for your personal development and academic performance over the past three months? | FU Usefulness |
| FU_USE_02 | Wie hilfreich war das Thema „Grit" in den letzten drei Monaten für Ihre persönliche Entwicklung und Ihre Schulleistungen? | How helpful was the topic "grit" for your personal development and academic performance over the past three months? | FU Usefulness |
| FU_USE_03 | Wie hilfreich war das Thema „Selbstdisziplin" in den letzten drei Monaten für Ihre persönliche Entwicklung und Ihre Schulleistungen? | How helpful was the topic "self-discipline" for your personal development and academic performance over the past three months? | FU Usefulness |
| FU_USE_04 | Wie hilfreich war das Thema „Selbstwirksamkeit" in den letzten drei Monaten für Ihre persönliche Entwicklung und Ihre Schulleistungen? | How helpful was the topic "self-efficacy" for your personal development and academic performance over the past three months? | FU Usefulness |
| FU_USE_05 | Wie hilfreich war das Thema „Resilienz" in den letzten drei Monaten für Ihre persönliche Entwicklung und Ihre Schulleistungen? | How helpful was the topic "resilience" for your personal development and academic performance over the past three months? | FU Usefulness |

*Note.* Same response format as Supplementary Table 1. The follow-up items mirror the posttest evaluation items but use a three-month retrospective framing: posttest items ask about anticipated applicability and usefulness, whereas follow-up items ask about applicability and usefulness as actually experienced over the preceding three months. Internal consistency in the present sample: α_FUA_ = .86, α_FUU_ = .91. Corresponds to Sections 3.2.2 and 4.2.3 of the main text.

**Supplementary Table 3***. Level 2 (Learning): Domain-Specific Knowledge Test Items*

| **No.** | **German** | **English** | **Key** |
| --- | --- | --- | --- |
| *GM1. Welche Aussagen entsprechen den Prinzipien eines Growth Mindset? / Which statements correspond to the principles of a growth mindset?* | | | |
| GM1_01 | Fähigkeiten sind genetisch festgelegt und unveränderlich. | Abilities are genetically determined and unchangeable. | F |
| GM1_02 | Fehler sind Gelegenheiten, um zu lernen. | Mistakes are opportunities to learn. | T |
| GM1_03 | Herausforderungen sollten vermieden werden, um Misserfolg zu vermeiden. | Challenges should be avoided in order to avoid failure. | F |
| GM1_04 | Intelligenz kann durch Übung und Lernen gestärkt werden. | Intelligence can be strengthened through practice and learning. | T |
| GM1_05 | Konstruktives Feedback ist eine Chance zur Verbesserung. | Constructive feedback is an opportunity for improvement. | T |
| *GM2. Welche neurobiologischen Mechanismen stehen mit einem Growth Mindset in Verbindung? / Which neurobiological mechanisms are associated with a growth mindset?* | | | |
| GM2_01 | Neuronenverbindungen können durch Lernen gestärkt werden. | Neural connections can be strengthened through learning. | T |
| GM2_02 | Neue neuronale Verbindungen entstehen bei wiederholter Übung. | New neural connections form through repeated practice. | T |
| GM2_03 | Intelligenz ist vollständig genetisch bedingt. | Intelligence is entirely genetically determined. | F |
| GM2_04 | Positive Selbstgespräche verbessern die neuronale Verarbeitung direkt. | Positive self-talk directly improves neural processing. | F |
| GM2_05 | Lernen kann die Speicherung von Informationen langfristig verbessern. | Learning can improve the long-term storage of information. | T |
| *Grit1. Welche der folgenden Elemente gehören zu den Säulen von Grit? / Which of the following elements are among the pillars of grit?* | | | |
| Grit1_01 | Hoffnung auf Erfolg. | Hope for success. | T |
| Grit1_02 | Tägliche Verbesserung durch Übung. | Daily improvement through practice. | T |
| Grit1_03 | Überwindung von Rückschlägen und Festhalten an Zielen. | Overcoming setbacks and holding on to one's goals. | T |
| Grit1_04 | Visualisierung erreichbarer Ergebnisse. | Visualizing attainable outcomes. | F |
| Grit1_05 | Leidenschaft für die eigenen Ziele entwickeln. | Developing passion for one's own goals. | T |
| *Grit2. Welche Strategien fördern Grit am effektivsten? / Which strategies promote grit most effectively?* | | | |
| Grit2_01 | Fehler betonen, damit sie stets präsent bleiben. | Emphasizing mistakes so that they remain constantly present. | F |
| Grit2_02 | Ein persönliches Lebensziel verfolgen, das langfristig Sinn stiftet. | Pursuing a personal life goal that provides long-term meaning. | T |
| Grit2_03 | Sich an Routineaufgaben halten, um schwierige Situationen zu vermeiden. | Sticking to routine tasks in order to avoid difficult situations. | F |
| Grit2_04 | Regelmäßige und anspruchsvolle Übungseinheiten einplanen, um dauerhaft besser zu werden. | Scheduling regular and demanding practice sessions in order to improve continuously. | T |
| Grit2_05 | Vor allem von anderen Unterstützung erwarten, damit Hindernisse leichter erscheinen. | Relying primarily on support from others so that obstacles appear easier. | F |
| *SD1. Ein 30-jähriger Unternehmensberater berichtet, dass er Schwierigkeiten hat, seine langfristigen beruflichen Ziele zu erreichen, da er häufig von kurzfristigen Aufgaben und Ablenkungen überwältigt wird. Er gibt an, dass ihm manchmal die Motivation fehlt, an Projekten kontinuierlich zu arbeiten. / A 30-year-old management consultant reports difficulty in reaching his long-term professional goals because he is frequently overwhelmed by short-term tasks and distractions. He states that he sometimes lacks the motivation to work on projects continuously.* | | | |
| SD1_01 | Planung und Priorisierung von Aufgaben auf Basis ihrer Wichtigkeit. | Planning and prioritizing tasks according to their importance. | T |
| SD1_02 | Entwicklung von Routinen, die den Arbeitsalltag klar strukturieren. | Developing routines that clearly structure the working day. | T |
| SD1_03 | Den Fokus auf leichte Aufgaben legen, um Frust zu meiden. | Focusing on easy tasks in order to avoid frustration. | F |
| SD1_04 | Vermeidung von Ablenkungen durch Zeitmanagement. | Avoiding distractions through time management. | T |
| SD1_05 | Delegation anspruchsvoller Aufgaben, um Stress zu reduzieren. | Delegating demanding tasks in order to reduce stress. | F |
| *SD2. Welche Strategien fördern langfristige Selbstdisziplin am effektivsten? / Which strategies promote long-term self-discipline most effectively?* | | | |
| SD2_01 | Ausschließlich Aufgaben mit geringem Schwierigkeitsgrad priorisieren. | Prioritizing exclusively tasks of low difficulty. | F |
| SD2_02 | Aufbau einer Erfolgsbilanz durch das Setzen kleiner erreichbarer Ziele. | Building a track record of success by setting small attainable goals. | T |
| SD2_03 | Routinen entwickeln, um Ablenkungen zu minimieren. | Developing routines in order to minimize distractions. | T |
| SD2_04 | Neue Vorhaben erst beginnen, wenn äußere Bedingungen perfekt erscheinen. | Beginning new undertakings only when external conditions appear perfect. | F |
| SD2_05 | Klare, realistische und messbare Ziele setzen, um Fortschritt sichtbar zu machen. | Setting clear, realistic, and measurable goals in order to make progress visible. | T |
| *SW1. Welche Faktoren fördern das Vertrauen in die eigene Selbstwirksamkeit? / Which factors promote confidence in one's own self-efficacy?* | | | |
| SW1_01 | Ein fernes Ziel ins Auge fassen, ohne Zwischenschritte zu berücksichtigen. | Focusing on a distant goal without considering intermediate steps. | F |
| SW1_02 | Positive Selbstgespräche, um Zweifel zu überwinden. | Positive self-talk in order to overcome doubts. | T |
| SW1_03 | Direkte Erfolge und der Umgang mit Rückschlägen. | Direct successes and the handling of setbacks. | T |
| SW1_04 | Eigene erfolgreiche Erfahrungen und die Reflexion über diese. | One's own successful experiences and reflection on them. | T |
| SW1_05 | Sich an Abläufe halten und neue Herausforderungen meiden. | Sticking to established procedures and avoiding new challenges. | F |
| *SW2. Ein 28-jähriger Berufseinsteiger berichtet über häufige Überforderung in neuen Projekten. Trotz fundierter Kenntnisse zweifelt er an seiner Fähigkeit, komplexe Probleme zu lösen. / A 28-year-old career entrant reports frequently feeling overwhelmed in new projects. Despite sound knowledge, he doubts his ability to solve complex problems.* | | | |
| SW2_01 | Bei Stress kurze Pausen einlegen, statt das eigentliche Problem anzugehen. | Taking short breaks when stressed instead of addressing the underlying problem. | F |
| SW2_02 | Regelmäßige Reflexion über Fortschritte und gezielte Konfrontation mit Herausforderungen. | Regular reflection on progress and deliberate confrontation with challenges. | T |
| SW2_03 | Suche nach spezifischem und konstruktivem Feedback, um die eigenen Leistungen besser einzuschätzen. | Seeking specific and constructive feedback in order to appraise one's own performance more accurately. | T |
| SW2_04 | Delegation aller schwierigen Aufgaben, um Fehler zu vermeiden. | Delegating all difficult tasks in order to avoid mistakes. | F |
| SW2_05 | Positives Feedback suchen, um das eigene Ansehen bei anderen zu steigern. | Seeking positive feedback in order to enhance one's standing with others. | F |
| *RES1. Welche Ansätze sind spezifisch geeignet, um Resilienz bei Rückschlägen aufzubauen? / Which approaches are specifically suited to building resilience in the face of setbacks?* | | | |
| RES1_01 | Vermeidung potenzieller Herausforderungen, um Belastungen zu reduzieren. | Avoiding potential challenges in order to reduce strain. | F |
| RES1_02 | Flexible Anpassung von Plänen an unvorhergesehene Herausforderungen. | Flexibly adapting plans to unforeseen challenges. | T |
| RES1_03 | Regelmäßige Reflexion über Rückschläge und Ableitung von Lernmöglichkeiten. | Regularly reflecting on setbacks and deriving learning opportunities from them. | T |
| RES1_04 | Visualisierung erreichbarer Ziele, um emotionale Stabilität zu fördern. | Visualizing attainable goals in order to foster emotional stability. | T |
| RES1_05 | Auf fremde Hilfen setzen, um Rückschläge abzugeben. | Relying on outside help in order to hand off setbacks. | F |
| *RES2. Eine 25-jährige Studentin berichtet, dass sie sich nach einer gescheiterten Prüfung schwer motivieren kann, weiterzulernen. Sie fühlt sich häufig emotional erschöpft und hat das Gefühl, ihre Ziele nicht erreichen zu können. / A 25-year-old university student reports that after failing an examination she finds it difficult to motivate herself to continue studying. She frequently feels emotionally exhausted and has the impression that she cannot reach her goals.* | | | |
| RES2_01 | Reflexion über bewältigte Rückschläge und die daraus gewonnenen Lernerfahrungen. | Reflecting on setbacks already mastered and the lessons learned from them. | T |
| RES2_02 | Aufbau eines unterstützenden sozialen Netzwerks, das emotionale und praktische Hilfe bietet. | Building a supportive social network that provides emotional and practical help. | T |
| RES2_03 | Vermeidung weiterer Rückschläge, indem schwierige Prüfungen verschoben werden. | Avoiding further setbacks by postponing difficult examinations. | F |
| RES2_04 | Nutzung von Entspannungsorten und Techniken zur mentalen Erholung. | Using restful settings and techniques for mental recovery. | T |
| RES2_05 | Direkte Belohnungen wählen, damit Motivation stabil bleibt. | Choosing immediate rewards so that motivation remains stable. | F |

*Note.* The knowledge test comprised ten item sets, each presenting five statements to be judged as applicable or not applicable, yielding 50 binary items. Item sets SD1, SW2, and RES2 presented a short case vignette instead of a direct question. Each statement was scored as correct when the participant's judgment matched the key; the total score was the proportion of correct judgments across all 50 statements. Because every statement required a dichotomous judgment, the theoretical chance level was .50. Key: T = statement applies and should be endorsed; F = distractor that should not be endorsed. Internal consistency in the present sample: α = .75, 95% CI [.63, .85]. Corresponds to Sections 3.2.1 and 3.4.1 of the main text.

**Supplementary Table 4.** *Level 1 (Reaction): One-Sample t Tests Against the Scale Midpoint*

| **Scale** | **n** | **M** | **SD** | **95% CI** | **t(40)** | **p** | **d** |
| --- | --- | --- | --- | --- | --- | --- | --- |
| Workshop satisfaction | 41 | 4.03 | 0.57 | [3.85, 4.21] | 11.64 | < .001 | 1.82 |
| Perceived application | 41 | 3.86 | 0.61 | [3.67, 4.06] | 8.98 | < .001 | 1.40 |
| Perceived usefulness | 41 | 3.87 | 0.65 | [3.67, 4.07] | 8.62 | < .001 | 1.35 |

*Note.* Items were rated on a 5-point Likert-type scale and tested against the neutral midpoint of 3. Corresponds to Section 4.2.1 of the main text.

**Supplementary Table 5.** *Level 3 (Behavior): Change From Posttest to Three-Month Follow-Up*

| **Scale** | **n** | **Posttest M (SD)** | **Follow-up M (SD)** | **Mean difference** | **95% CI** | **V** | **p (Wilcoxon)** | **t(16)** | **p (t test)** | **d_z** |
| --- | --- | --- | --- | --- | --- | --- | --- | --- | --- | --- |
| Perceived application | 17 | 4.02 (0.51) | 2.27 (0.78) | −1.75 | [−2.12, −1.37] | 0 | < .001 | −9.97 | < .001 | −2.42 |
| Perceived usefulness | 17 | 3.91 (0.63) | 2.68 (0.89) | −1.23 | [−1.61, −0.85] | 0 | < .001 | −6.87 | < .001 | −1.67 |

*Note.* Wilcoxon signed-rank tests were the primary inferential procedure; paired-samples t tests are reported as a robustness check. Posttest values refer to the n = 17 participants with complete follow-up data and therefore differ from the full posttest sample in Supplementary Table 4. The posttest and follow-up versions of these scales differed in framing (see Supplementary Table 2), so these changes cannot be attributed to declining transfer alone. Corresponds to Section 4.2.3 of the main text.

**Supplementary Table 6.** *Selective Attrition: Participants With Versus Without Follow-Up Data*

| **Variable** | **Follow-up M (SD)** | **n** | **No follow-up M (SD)** | **n** | **t** | **df** | **p** | **Hedges' g** |
| --- | --- | --- | --- | --- | --- | --- | --- | --- |
| Workshop satisfaction | 4.02 (0.53) | 17 | 4.04 (0.60) | 24 | −0.10 | 37.0 | .920 | −0.03 |
| Perceived application | 4.02 (0.51) | 17 | 3.75 (0.67) | 24 | 1.47 | 38.8 | .150 | 0.44 |
| Perceived usefulness | 3.91 (0.63) | 17 | 3.84 (0.67) | 24 | 0.34 | 36.1 | .734 | 0.11 |
| Knowledge test score | 0.88 (0.08) | 17 | 0.86 (0.09) | 24 | 0.84 | 35.4 | .408 | 0.26 |

*Note.* Welch two-sample t tests. Positive values indicate higher scores among participants who provided follow-up data. This comparison is itself based on small subgroups and is not sensitive to small or moderate differences; a null result therefore does not rule out selective attrition. Corresponds to Section 4.2.3 of the main text.

**Supplementary Table 7.** *Level 4a (Results): Academic Grades Across the Two School Terms*

*Panel A. Descriptive statistics*

| **Group** | **n** | **Term 1 M (SD)** | **95% CI** | **Term 2 M (SD)** | **95% CI** | **Change M** | **95% CI** |
| --- | --- | --- | --- | --- | --- | --- | --- |
| Experimental | 41 | 10.64 (1.87) | [10.05, 11.23] | 10.95 (1.97) | [10.33, 11.57] | 0.30 | [0.13, 0.48] |
| Control | 46 | 10.12 (1.68) | [9.62, 10.62] | 10.37 (1.88) | [9.81, 10.93] | 0.26 | [0.05, 0.46] |

*Panel B. Mixed-design analysis of variance*

| **Effect** | **F** | **df** | **p** | **η²p** |
| --- | --- | --- | --- | --- |
| Time | 17.05 | 1, 85 | < .001 | .167 |
| Group | 1.99 | 1, 85 | .162 | .023 |
| Time × Group | 0.12 | 1, 85 | .725 | .001 |

*Panel C. Linear mixed-effects model and change-score analysis*

| **Analysis** | **Estimate / statistic** | **95% CI** | **df** | **p** |
| --- | --- | --- | --- | --- |
| LMM: effect of time | 0.26 | [0.07, 0.44] | 85.0 | .007 |
| LMM: time × group | 0.05 | [−0.22, 0.31] | 85.0 | .725 |
| Change-score comparison | t = −0.36 | [−0.31, 0.22] | 84.4 | .722 |
| Change-score effect size | Hedges' g = −0.08 | — | — | — |

*Note.* Grades are reported on the German 0 to 15 point scale, with higher values indicating better performance. Levene's tests indicated homogeneity of variances at both time points (Term 1: F(1, 85) = 0.17*, p* = .683; Term 2: F(1, 85) = 0.01, *p* = .913). The groups did not differ significantly at baseline, *t*(81.0) = −1.38, *p* = .173, Hedges' *g* = −0.29. The linear mixed-effects model included random intercepts for students; the control group served as the reference category. In the baseline and change-score comparisons, negative values indicate lower scores in the control group. Corresponds to Section 4.2.4 of the main text.

**Supplementary Table 8.** *Level 4b (Results): Wilcoxon Signed-Rank Robustness Checks for the Five Target Constructs*

| **Construct** | **n** | **V** | **p** | **p (Holm)** | **r** |
| --- | --- | --- | --- | --- | --- |
| Growth mindset | 17 | 96.0 | .153 | .460 | .35 |
| Grit | 17 | 68.5 | .329 | .659 | .24 |
| Self-discipline | 17 | 119.0 | .047 | .233 | .48 |
| Self-efficacy | 17 | 116.0 | .064 | .258 | .45 |
| Resilience | 17 | 51.5 | .407 | .659 | .20 |

*Note.* Non-parametric robustness checks for the paired-samples t tests reported in Table 2 of the main text, based on the *n* = 17 participants with complete pre-follow-up data on all five constructs. V is the sum of positive ranks; non-integer values result from ties. *p* values were adjusted using Holm's sequential procedure. The effect size r was computed as z divided by the square root of n. No construct reached statistical significance after correction. Corresponds to Section 4.2.5 of the main text.
